# WNK Signaling Is Involved in Neural Development via Lhx8/Awh Expression

**DOI:** 10.1371/journal.pone.0055301

**Published:** 2013-01-30

**Authors:** Atsushi Sato, Hiroshi Shibuya

**Affiliations:** Department of Molecular Cell Biology, Medical Research Institute, Tokyo Medical and Dental University, Bunkyo-ku, Tokyo, Japan; Simon Fraser University, Canada

## Abstract

WNK kinase family is conserved among many species and regulates SPAK/OSR1 and ion co-transporters. Some mutations in human WNK1 or WNK4 are associated with Pseudohypoaldosteronism type II, a form of hypertension. WNK is also involved in developmental and cellular processes, but the molecular mechanisms underlying its regulation in these processes remain unknown. Here, we identify a new target gene in WNK signaling, *Arrowhead* and *Lhx8*, which is a mammalian homologue of *Drosophila* Arrowhead. In *Drosophila*, *WNK* was shown to genetically interact with *Arrowhead*. In *Wnk1* knockout mice, levels of *Lhx8* expression were reduced. Ectopic expression of *WNK1*, *WNK4* or *Osr1* in mammalian cells induced the expression of the *Lhx8*. Moreover, neural specification was inhibited by the knockdown of both *Wnk1* and *Wnk4* or *Lhx8*. *Drosophila WNK* mutant caused defects in axon guidance during embryogenesis. These results suggest that WNK signaling is involved in the morphological and neural development via Lhx8/Arrowhead.

## Introduction

WNK (with no lysine (K)) is a family of serine/threonine protein kinases that are characterized by an atypical location of the catalytic lysine and are conserved among many species, such as plants, nematode, fly, rat, mouse and human [1]–[3]. There are four mammalian WNK family members, and positional cloning has identified two of them, WNK1 and WNK4, as genes linked to a hereditary form of human hypertension known as Pseudohypoaldosteronism type II (PHAII) [4]. Several groups including our group previously discovered that WNK1 and WNK4 could phosphorylate and activate SPAK or OSR1 kinases, which in turn regulates various ion co-transporters, such as NKCC1, NKCC2 and NCC [5]–[8]. We also found that dysregulation of WNK1 and WNK4 in mouse kidney caused phenotypes similar to those of PHAII [9]. These results suggest that the dysregulation of sodium and potassium transport by WNK1 and WNK4 contribute to the pathogenesis of hypertension in PHAII patients.

WNK family members have also been identified in screens of cultured cells for enhanced cell survival and proliferation [10]. WNK1 is required for cell division in cultured cells [11], and proliferation, migration and differentiation of neural progenitor cells [12]. In addition, *Wnk1* is ubiquitously expressed in mice, and knockout of the gene is lethal before embryonic day 13 (Zambrowicz et al. and in this report) [13], with the developing mice displaying defects in cardiac development [14]. Furthermore, PHAII patients exhibit other clinical problems in addition to hypertension, such as an intellectual impairment, dental abnormalities and impaired growth [15]. The *Drosophila* genome contains a single WNK gene (called as CG7177 in Flybase (http://flybase.org)), which was identified in screens for genes involved in cell cycle or neural development [10], [16]. These observations suggest that WNK1 plays unknown roles in developmental processes, in addition to its control of ion co-transporters in the kidney.

Here, we demonstrate that the functions of the WNK signaling pathway are conserved between mammals and flies. Mutation of *Drosophila WNK (DWNK)* caused several morphological defects. Our functional analysis of *DWNK* identified a new target gene, Arrowhead (Awh), and we found that the mammalian homologue of Awh, Lhx8, is also a target gene of the WNK signaling pathway in mammalian cells. Furthermore, we demonstrated that the WNK signaling pathway modulates *Drosophila* development via Awh, and modulates neural specification in mammalian cells via Lhx8. These results reveal a novel role for WNK signaling via Lhx8 or Awh in the regulation of morphological and neural development.

## Materials and Methods

### Ethics statement

All animal experiments were performed under the ethical guidelines of Tokyo Medical and Dental University, and animal protocols were reviewed and approved by the animal welfare committee of the Tokyo Medical and Dental University.

### Fly stocks and genetics

Fly strains used in this study were; Canton-S, *yw*, EY10165 (UAS-*DWNK*; Bloomington Stock Center), *fray^07551^*, *Awh^63Ea-E12^*, *hh-Gal4*, *sd*-*Gal4*, *da-Gal4* and *1407-Gal4* (Bloomington Stock Center). Flies with UAS-*DWNK*, UAS-*DWNK^D420A^*, UAS-*hWNK1*, UAS-*mOsr1*, UAS-*fray*, UAS-*fray^S347D^* and UAS-*Awh* were generated by P-mediated germline transformation (injected by BestGene Inc.).

*DWNK^EY18^*, a null mutation of *DWNK*, is a derivative of EY10165. *DWNK^EY18^* has a 1712 bp deletion from the EY10165 insertion point to the middle of exon 3, which includes the translation start site (red line in Fig. S1). However, the 5′ region of the P element of EY10165 was retained (1365 bp). We confirmed by RT-PCR analysis that *DWNK^EY18^* produced truncated transcripts, by the presence of several poly-A signal sequences in the retained P element sequences (* in Fig. S1; data not shown).

Genotypes of all fly lines we used in this study were in figure legends. We used *yellow* transgene for the clonal marker; the wild type body color represents heterozygous tissue, and *yellow* body color represents mutant tissue. The mutant tissues were judged by discrimination of the light color compared with the background of wild type color, and the clonal borders were shown by thin black lines.

### Molecular cloning

Based on the predicted amino acid sequence of CG7177, we confirmed the intron-exon junctions of *DWNK* by RT-PCR. *Lhx8* cDNA for *in situ* probe was obtained by RT-PCR. *fray* (RE53265) and *Awh* (RE24382) cDNA clones were obtained from Drosophila Genomics Resource Center (Indiana, USA). *Lhx8*, *Lhx6* and *Isl1* cDNAs for the rescue experiments were obtained by RT-PCR. To construct the kinase-dead form of *DWNK* and *fray*, and the constitutive active of *fray*, we performed site-directed mutagenesis, using the following primers: for *DWNK^D420A^*, 5′- GTTAAAATCGGCGCCTTGGGCCTGG -3′ and 5′- CCAGGCCCAAGGCGCCGATTTTAAC -3′; for *fray^K67M^* 5′- GAAGTGCGCCATTATGCGCATCAACCTGG -3′ and 5′- CCAGGTTGATGCGCATAATGGCGCACTTC -3′; for *fray^S347D^* 5′- CCAACCAGGAGCCGACGGCCGTTTGCATC -3′ and 5′- GATGCAAACGGCCGTCGGCTCCTGGTTGG -3′.

### *In vitro* kinase assay

HEK293T cells were transfected with Flag-*DWNK*, Flag-*DWNK^D420A^*, Flag-*Fray*, Flag-*Fray^K67M^* or Flag-*Fray^S347D^* expression plasmids. The lysates were prepared from transfected cells and immunoprecipitated with anti-Flag M2 antibody (Sigma). Immunoprecipitates were incubated with bacterially expressed GST fusion proteins (GST-Fray^K67M^ or GST-NCC) in kinase buffer containing 10 mM HEPES (pH 7.4), 1 mM DTT, 5 mM MgCl_2_, and 5 µCi of [γ-^32^P]-ATP at 30°C. Phosphorylated substrates were subjected to SDS-PAGE, detected by an image analyzer FLA3000 (Fujifilm) and quantified by Multi Gauge software (GE).

### Antibodies

Antibodies used in this report were; mouse anti-Flag M2 (Sigma), rabbit anti-Flag (Sigma), mouse anti-HA (Cell signaling), rat anti-HA (Roche), mouse anti-T7 (Merck), rabbit anti-T7 (MBL), rabbit anti-OSR1 [5], rabbit anti-phospho-OSR1 [5], anti-mouse HRP conjugated (GE), anti-rabbit HRP conjugated (GE), anti-rat HRP conjugated (GE), anti-digoxigenin alkaline phosphatase conjugated (Roche), mouse monoclonal antibody 22C10 (DSHB), rabbit anti-LacZ (Cappel; used for the selection of Balancer chromosomes), rabbit anti-GFP (MBL), anti-rabbit IgG AlexaFluor 488 conjugated (Invitrogen) and anti-mouse IgG Cy3 conjugated (Jackson) antibodies.

### Histology and staining

All wings were mounted in GMM [17]. Antibody staining, *In situ* hybridization to fly and mouse embryos were carried out as described previously [18], [19]. For *in situ* hybridization, digoxigenin-labeled RNA probes were prepared by *in vitro* transcription using *Awh* and *Lhx8* cDNA as a template. Images were obtained using SteREO Discovery, Axioscope and Axio Observer (Carl Zeiss), and processed using Axiovision with extended focus (Carl Zeiss) and Photoshop (Adobe).

### *Wnk1* knockout mouse and microarray

The *Wnk1* knockout mouse was generated by a gene-trap insertion. The primers for genotyping of *Wnk1* knockout mice were the following: OYC4-WT 5′- AAAATACTCTGTCAGGCTTAAGTGT -3′ for wild-type, LTR2 5′- AAATGGCGTTACTTAAGCTAGCTTGC -3′ for the *Wnk1* mutant and OYC4-3′ 5′- TGAAGCCAGGCATTAAGCACTC -3′ was used as a common primer.

We isolated total RNA using RNeasy kit (Qiagen). Microarray was performed by Takara-Bio using GeneChip (Affymetrix).

### Culture cell lines

Cell lines used in this study were; HEK293T, NIH3T3 and Neuro2A [20]. The growth medium for HEK293T cells and NIH3T3 cells was DMEM with 10% FBS, and for Neuro2A cells, DMEM with 20% FBS. For transfection, we used Lipofectamine 2000 (Invitrogen) or polyethylenimine (Polysciences) for plasmids and Lipofectamine RNAiMax (Invitrogen) for siRNA. The target sequence of siRNA against mouse *WNK1* was 5′- GAUAGGGUGUCCUUAAUUA -3′, against mouse *WNK4* was 5′- GAAAUCGAGGACUUAUACA -3′, and against mouse *Lhx8* was 5′- AGAAUAAGCCAUUUCUUCC -3′. For the hypertonic treatments, we used serum-free DMEM with 500 mM Sorbitol for long incubation and hypertonic buffer for short incubation. Hypertonic buffer contained 130 mM NaCl, 2 mM KCl, 2 mM CaCl2, 2 mM MgCl_2_, 1 mM KH_2_PO_4_, 10 mM Glucose, 10 mM Sodium HEPES (pH 7.4) and 520 mM Sorbitol. For the differentiation of Neuro2A cells, we used serum-free DMEM with 10 µM retinoic acid for 24 hours induction or DMEM with 1% FBS and 10 µM retinoic acid for 48 hours induction.

### RT-PCR analysis

Total RNA was isolated by TRIzol (Invitrogen). cDNA synthesis was carried out using Moloney murine leukemia virus reverse transcriptase (Invitrogen). The reaction mixture were denatured at 94 degree for 5 minutes and then cycled at 98 degree/15 seconds and 72 degree/30 seconds, then followed by a final 3 minutes extension at 72 degree (for *mouse Wnk1*, *mouse Wnk4*, *human WNK1*, *human WNK4*, *mouse Osr1*, *mouse Choline acetyltransferase* (*ChAT*), *mouse Glutamic acid decarboxylase 1* (*Gad1*), *mouse Lhx6* and *mouse Glyceraldehyde-3-phosphate dehydrogenase* (*GAPDH*)), or at 94 degree for 5 minutes and then cycled at 98 degree/15 seconds, 58 degree/15 seconds and 72 degree/30 seconds, then followed by a final 3 minutes extension at 72 degree (for *mouse Lhx8* and *mouse Islet-1* (*Isl1*)). Numbers of cycles are depending on samples (see below, number of cycles are shown after primer sequences). *GAPDH* was used for normalization of cDNA samples. The sequences of the primer pairs for PCR were as follows: mouse *Wnk1*, 5′- AGAGGATGGCTCAGGTAGTCCACAC -3′ and 5′- AACACACAGCTGCCCAGGAGCAGAG -3′ (29 cycles), mouse *Wnk4*, 5′- AAGCTCTGGCTGCGCATGGAGGATG -3′ and 5′- GGATCGAGGTCTCCGTCGAAGAGTC -3′ (32 cycles), human *WNK1*, 5′- AAGTTAGAGCTGCGACGACTACGAG -3′ and 5′- GGTGCAGAGAACTTCCTTGCCATTC -3′ (25 cycles), human *WNK4*, 5′- CCAAGTGACTTCATCCAAGGAACCG -3′ and 5′- TCAGAGAGTTCCTTCGCATGATGCC -3′ (25 cycles), mouse *Osr1*, 5′- TGGCCGTCTCCATAAGACAGAGGAC -3′ and 5′- TATCCGAGCCTTCAACACCAGATGC -3′ (24 cycles), mouse *Lhx8*, 5′- GACCCAGCTGCCAATAAGTCATACC -3′ and 5′- GACACACACTCGAGCCAACTATCTC -3′ (35 cycles), mouse *ChAT*, 5′- CAGTGCATGCAACACCTGGTACCTG -3′ and 5′- GAACAGATCACCCTCACTGAGACGG -3′ (45 cycles), mouse *Gad1*, 5′- CATCTTCCACTCCTTCGCCTGCAAC -3′ and 5′- CAGTCAACCAGGATCTGCTCCAGAG -3′ (40 cycles), mouse *Lhx6*, 5′- CACTCTGCGCCTCTCTTCGCACTGC -3′ and 5′- ATGTGCGACACACGGAGCACTCGAG -3′ (36 cycles), mouse *Isl1*, 5′- ACATCGAGTGTTTCCGCTGTGTAGC -3′ and 5′- CTACTGGGTTAGCCTGTAAACCACC -3′ (28 cycles) and mouse *GAPDH*, 5′- GCCATCACTGCCACCCAGAAGACTG -3′, and 5′- CATGAGGTCCACCACCCTGTTGCTG -3′ (21 cycles). The whole gel images of all PCR results are shown in Fig. S9.

### Quantification and statistical analysis

All results from Western blotting were quantified using Multi Gauge software (GE). Quantitative PCR was performed with an Applied Biosystems 7300 Real-Time PCR Cycler (ABI) using THUNDERBIRD SYBR qPCR Mix (TOYOBO). The sequences of the primer pairs for *Lhx8*, *ChAT*, *Gad1* and *GAPDH* were described previously [21]–[23]. *GAPDH* was used for normalization of cDNA samples. Data are computed using Microsoft Exel (Microsoft). Values and error bar represent the mean and SD and are representative of at least 3 independent experiments.

## Results

### WNK- SPAK/OSR1 pathway is conserved between mammals and flies

**Figure 1:**
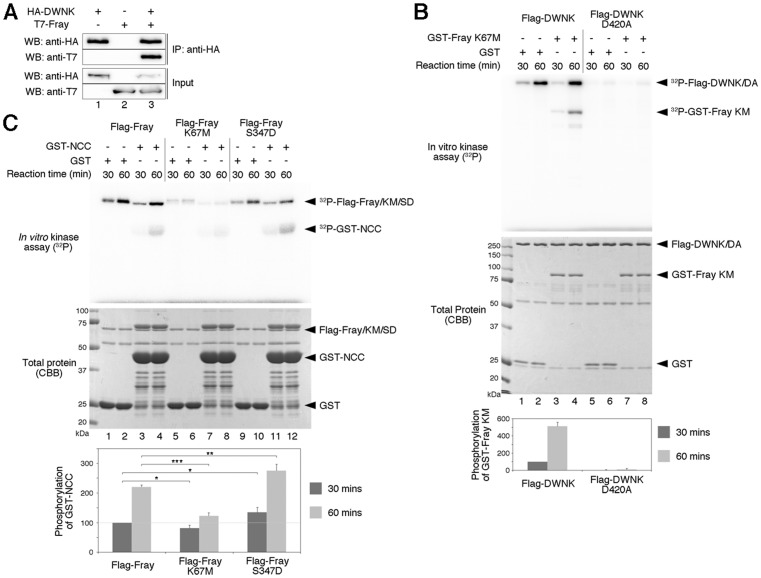
DWNK directly binds to and phosphorylates Fray, and S347D mutation of Fray caused the constitutive activation. (A) Interaction between DWNK and Fray examined by co-immunoprecipitation. Immunoprecipitates (IP) were subjected to Western blotting (WB) with the indicated antibodies. +, present; −, absent. (B) Phosphorylation of Fray by DWNK. Upper panel showed the result of *in vitro* kinase assay. Upper bands (^32^P-Flag-DWNK/DA) represent the auto-phosphorylation of DWNK. Lower bands represent the phosphorylation of Fray (^32^P-GST-FrayKM). We used the kinase-dead form of Fray (Fray^K67M^) for avoiding an auto-phosphorylation of Fray itself. DWNK could phosphorylate Fray, but the kinase-dead form of DWNK (DWNK^D420A^) could not. Lower panel showed the total protein using *in vitro* kinase assay. The bar graph showed normalized relative kinase activity. The value of DWNK at 30 minutes was set to 100. (C) Phosphorylation of truncated NCC by Fray. Upper panel showed the result of *in vitro* kinase assay. Upper bands (^32^P-Flag-Fray/KM/SD) represent the auto-phosphorylation of Fray. Lower bands represent the phosphorylation of NCC (^32^P-GST-NCC). Fray could phosphorylate NCC, but the kinase-dead form of Fray, Fray^K67M^, could not. Phosphorylation by Fray^S347D^ was stronger by Fray, indicating that Fray^S347D^ is a constitutively active form of Fray. Lower panel showed the total protein using *in vitro* kinase assay. The bar graph showed normalized relative kinase activity of 3 independent experiments. The value of Fray at 30 minutes was set to 100. Statistical significance was determined by Student's *t*-test; *P<0.1, **P<0.05, ***P<0.01.

The *Drosophila* genome contains only one WNK homologue CG7177, which we hereafter refer to as *Drosophila* WNK (DWNK; Fig. S1). In contrast, there are four WNK family genes in mammals. Among the mammalian WNK proteins, DWNK was most homologous to WNK1 (Fig. S1). Recent studies have shown that mammalian WNK1–4 interact with and phosphorylate the STE20 kinases, SPAK and OSR1 [5]–[7]. As *Drosophila* Fray is a homologue of SPAK and OSR1 [24], we investigated whether the biochemical interaction between DWNK and Fray is conserved in *Drosophila*. We transiently expressed HA-DWNK together with T7-Fray in human embryonic kidney (HEK) 293T cells. When cell extracts were subjected to immunoprecipitation with the HA antibody, followed by immunoblotting, we found that DWNK interacted with Fray (Fig. 1A, lane 3). We next investigated phosphorylation of Fray by DWNK. We produced a glutathione S-transferase (GST)-tagged kinase-negative form of Fray^K67M^ in bacteria and tested its ability to be phosphorylated *in vitro*. We observed that DWNK phosphorylated Fray in a kinase-dependent manner (Fig. 1B, lanes 4, 8). We attempted to generate a constitutively activate form of Fray by mutating Ser to Asp at amino acid 347, which corresponds to the site of WNK phosphorylation in mouse Osr1 [5], [6]. Fray kinase activity was assayed using an amino-terminal fragment of NCC, GST-NCC, as a substrate [5]. This mutant, Fray^S347D^, exhibited increased phosphorylation of GST-NCC, relative to wild-type Fray (Fig. 1C lane 4,12), indicating that the mutation of Ser-347 to Asp causes constitutive activation of Fray.

**Figure 2:**
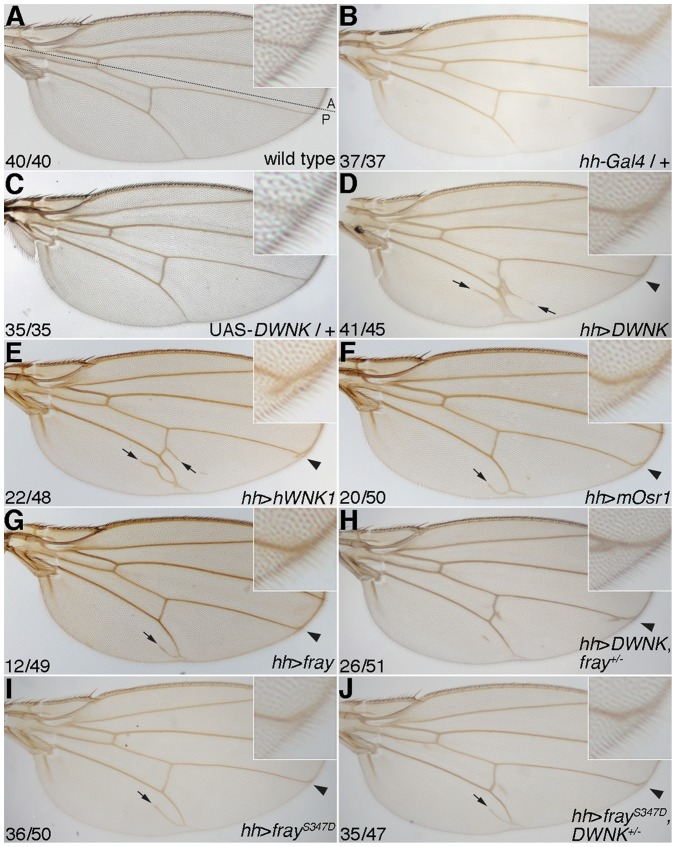
Conservation of the WNK-OSR1 pathway. (A) Wild-type wing. Dotted line indicates the anterior (A)-posterior (P) boundary. (B–C) Wings from heterozygous *hedgehog* (*hh*)-*Gal4* (B) or UAS-*DWNK* (C). Both wings did not show any phenotype. (D–G) Wings from EY10165 (UAS-*DWNK*) (D), UAS-*hWNK1* (E), UAS-*mOsr1* (F) and UAS- *fray* (G) flies driven by *hh-Gal4*. Additional vein around vein 5 (arrow) and delta phenotype at vein 4 (arrowhead) were observed. (H) Wing from flies overexpressing *DWNK* driven by *hh*-*Gal4* with *fray^07551^* heterozygous mutant. (I–J) Wings from *fray^S347D^* overexpression flies driven by *hh-Gal4* without (I) or with (J) *DWNK^EY18^* heterozygous mutant. The numbers of wings showing the phenotypes and of total observed wings were indicated. Anterior is up. Distal is right. Insets of upper right corner show the magnification around vein 4. The detail genotypes in this figure were followings: (A) Canton-S (wild type): (B) *y w hsflp*/*y w*; *hh-Gal4*/+: (C) *y w*; EY10165/+: (D) *y w*; *hh-Gal4*/EY10165: (E) *y w hsflp*/*y w*; *hh-Gal4*/UAS-*hWNK1*: (F) *y w hsflp*/y w; UAS-*mOSR1*/+; *hh-Gal4*/+: (G) *y w hsflp*/*y w*; UAS-*fray*/+; *hh-Gal4*/+: (H) *y w hsflp*/*y w*; *hh-Gal4* EY10165/*fray^07551^*: (I) *y w hsflp*/*y w*; *hh-Gal4*/UAS-*fray* ^S347D^: (J) *y w hsflp*/*y w*; *hh-Gal4* UAS-*fray* ^S347D^/*DWNK^EY18^*. See also Fig. S2.

To examine the functional conservation of human and *Drosophila WNK*, we used the UAS-Gal4 system to express human *WNK* in *Drosophila*. We made an expression construct of human *WNK1* (*hWNK1*) and generated UAS-transgenic flies (UAS-*hWNK1*). As a UAS-*DWNK* line, we used the EY10165 line, in which the pEY construct has been inserted into the 1^st^ exon of the *DWNK* gene (Fig. S1B). Although any wing phenotypes were not obtained from either the heterozygous UAS-*DWNK* or *hedgehog* (*hh*)-*Gal4* driver line (Fig. 2B–C compared with Fig. 2A, and see also Fig. S2), we observed that the phenotypes of *DWNK* overexpression using *hh-Gal4* driver were similar to those of *hWNK1* overexpression (Fig. 2D–E compared with Fig. 2A; extra veins around vein 5 and delta phenotypes at vein 4 in the wing; see also Fig. S2). We also generated UAS-transgenic flies of mouse *Osr1* (*mOsr1*; UAS-*mOSR1*) and its fly homologue, *fray* (UAS-*fray*) [24]. Wing phenotypes of the flies overexpressing *mOsr1* were similar to those overexpressing *fray* (Fig. 2F–G and Fig. S2). To analyze the genetic interaction between DWNK and Fray, we examined the phenotypes of *DWNK* overexpression in a heterozygous *fray* mutant (*fray^07551^*) background. Heterozygous *fray^07551^* has a normal-looking wing. The penetrance of wing phenotypes induced by *DWNK* overexpression were partially rescued in a heterozygous *fray^07551^* mutant background (Fig. 2H and Fig. S2). Moreover, the wing phenotypes induced by *fray^S347D^*, the constitutively active form of Fray driven by *hh-Gal4*, were more frequent, but similar phenotypes to those seen in flies overexpressing *mOsr1*or *fray* (Fig. 2I and Fig. S2). We also generated an inactive mutant of *DWNK*, *DWNK^EY18^*, by imprecise P element excision (Fig. S1; see also Materials and Methods for detail characterization of *DWNK* mutant). Heterozygous *DWNK^EY18^* mutant did not cause any phenotype in wing. The penetrance of the phenotypes of *fray^S347D^* overexpression could not be rescued in a heterozygous *DWNK^EY18^* background (Fig. 2J and Fig. S2). Taken together, these results suggest that Fray functions downstream of DWNK, and that the WNK-SPAK/OSR1/Fray pathway is conserved among many species.

### DWNK-Fray pathway functions in abdominal development

**Figure 3:**
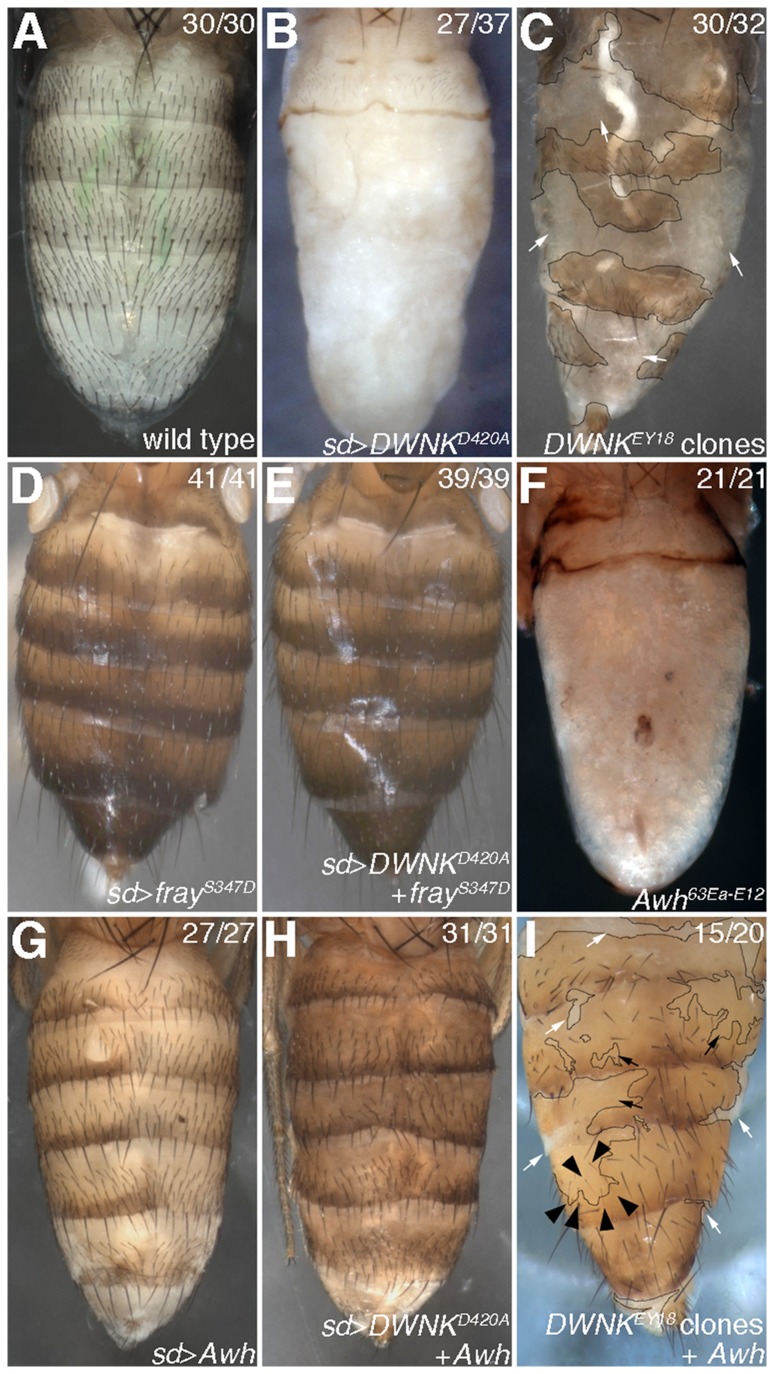
Awh is a downstream target of DWNK. (A) Abdomen from wild-type pharate adult. (B) Abdomen from pharate adult overexpressing *DWNK^D420A^* driven by *sd*-*Gal4*. (C) Abdomen from pharate adult with *DWNK^EY18^* minute clones (white arrows). Thin black lines indicate the clone border. (D) Abdomen from adult overexpressing *fray^S347D^* driven by *sd*-*Gal4*. (E) Abdomen from adult overexpressing both *DWNK^D420A^* and *fray^S347D^* driven by *sd*-*Gal4*. (F) Abdomen from *Awh^63Ea-E12^* pharate adult. (G) Abdomen from pharate adult overexpressing *Awh* driven by *sd*-*Gal4*. (H) Abdomen from pharate adult overexpressing both *DWNK^D420A^* and *Awh* driven by *sd*-*Gal4*. (I) Abdomen from adult with *DWNK^EY18^* minute clones and *Awh* overexpression. *Awh* was expressed only in *DWNK^EY18^* minute clones using the *Gal80* suppression technique. Thin black lines indicate the clone border (also *Awh* expression area). White arrows show the remaining abdominal defects. Black arrows or black arrowheads show rescued abdominal cuticles or bristles, respectively. The numbers of abdomens showing the phenotypes and of total observed abdomen were indicated. Dorsal views. Anterior is up. The detail genotypes in this figure were followings: (A) Canton-S (wild type): (B) *w sd-Gal4*/+; UAS-*DWNK^D420A^*/+: (C) *y w hsflp*; *DWNK^EY18^* FRT2A/*hsGFP hsCD2*(*y^+^*) *M(3)i55 ri* FRT2A: (D) *w sd-Gal4*/+; UAS-*fray^S347D^*/+: (E) *w sd-Gal4*/+; UAS-*DWNK^D420A^*/+; UAS-*fray^S347D^*/+: (F) *y w hsflp*; *Awh^63Ea-E12^*: (G) *w sd-Gal4*/+; UAS-*Awh*/+: (H) *w sd-Gal4*/+; UAS-*DWNK^D420A^*/+; UAS-*Awh*/+: (I) *y w hsflp*; *arm*-*Gal4*/UAS-*Awh*; *DWNK^EY18^* FRT2A/*hsGFP hsCD2*(*y^+^*) *M(3)i55 Tub>Gal80* FRT2A.

To investigate the developmental function of DWNK, we generated UAS system constructs for the expression of the kinase-dead form of *DWNK* (UAS-*DWNK^D420A^*). As the expression of *DWNK^D420A^* by *hh-Gal4* caused lethal, we switched to *sd-Gal4* driver. The expression of *DWNK^D420A^* by *sd-Gal4* driver induced the loss of wing margin (Fig. S3). In addition, the expression of *DWNK^D420A^* by *sd-Gal4* driver caused the complete disruption of abdominal differentiation in the pharate adults (compared Fig. 3B with Fig. 3A). To confirm that these phenotypes were caused by the loss of *DWNK*, we examined the adult phenotypes of *DWNK^EY18^*. We employed a mosaic analysis since homozygous *DWNK^EY18^* flies die between late embryonic stage and early 2^nd^ instar larva. Various phenotypes, such as extra veins in the wing and loss of macrocheate or microcheate bristles in the notum, were observed in the large *DWNK^EY18^* clones generated by *Minute* methods (Fig. S3). Moreover, the defects in abdominal development were observed in the mosaic clones (Fig. 3C). We also observed that the abdominal phenotypes were almost rescued by the overexpression of *DWNK* (Fig. S4), suggesting that the abdominal phenotypes were the results of *DWNK* deficiency. These observations indicate that phenotypes induced by the kinase-dead form of DWNK, DWNK^D420A^, were indeed caused by loss of DWNK function, suggesting that DWNK^D420A^ worked as a dominant negative.

Next, we examined whether Fray also worked at the downstream of WNK in abdominal development. While the expression of the constitutively active form of *fray*, *fray^S347D^*, did not cause any phenotype in abdomen (Fig. 3D), the abdominal defects caused by *DWNK^D420A^* could be rescued by the expression of *fray^S347D^* (Fig. 3E). We also confirmed that this rescue was not due to the titration of *Gal4* expression by adding another UAS construct (Fig. S5). Thus, these results suggest that DWNK-Fray pathway plays important roles in *Drosophila* abdominal development.

### Genetic interaction of DWNK with Awh

Both the *DWNK* minute clones and flies expressing the dominant-negative form of *DWNK* exhibited defects in abdominal development (Fig. 3B–C). Similar abdominal phenotypes have been previously described for the *Arrowhead* (*Awh*) mutant (Fig. 3F) [25], a transcription factor of the Lim homeobox type. We therefore investigated whether *DWNK* genetically interacts with *Awh*. We generated UAS-*Awh* lines and induced *Awh* expression by *sd-Gal4* driver, together with or without *DWNK^D420A^* expression. Overexpression of *Awh* did not affect the abdominal development (Fig. 3G). However, the defect in abdominal development caused by expression of *DWNK^D420A^* was rescued in flies co-expressing *Awh* (Fig. 3H and 3B). We also tested whether expression of *Awh* rescued the abdominal phenotype of *DWNK* minute clones. Using a combination of the FLP/FRT mosaic system and Gal80 suppression, we could express *Awh* locally in *DWNK* minute clones. As shown in Fig. 3I, overexpression of *Awh* partially rescued the abdominal phenotype of *DWNK* minute clones. These results indicate that DWNK genetically interacts with Awh.

### WNK regulates the expression of the Awh/Lhx8 gene

**Figure 4:**
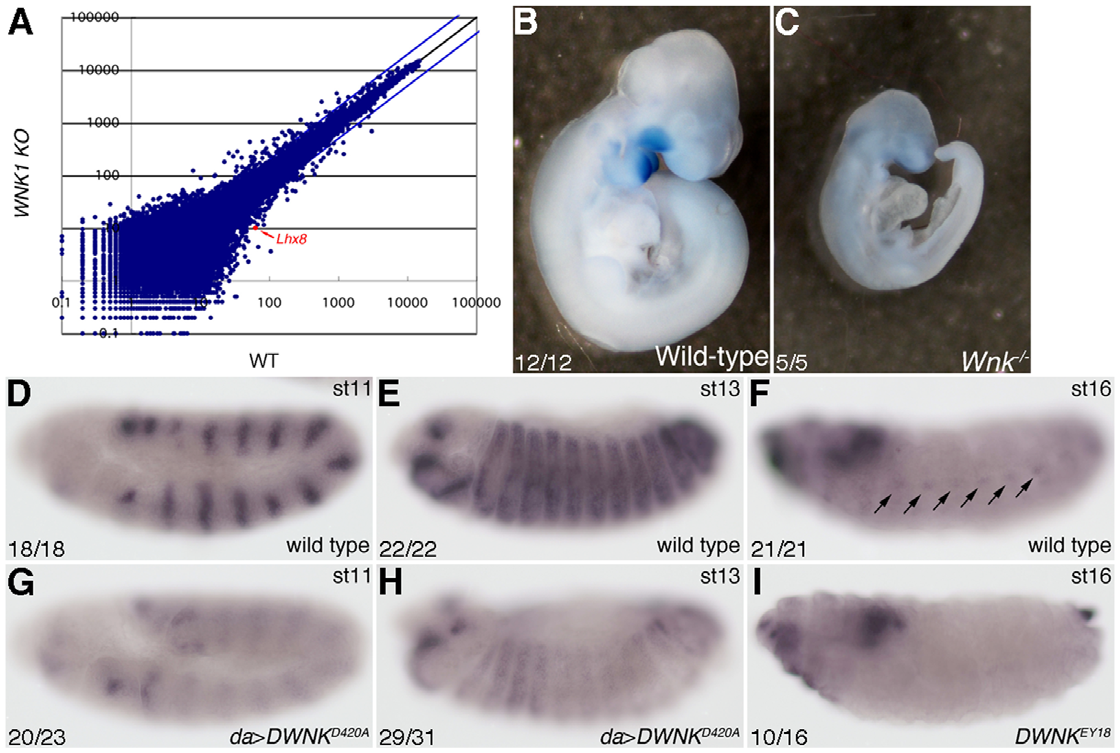
WNK/DWNK regulate the expression of *Lhx8*/*Awh*. (A) Microarray data from *Wnk1* knockout mice at E9.5 compared with wild-type mice. *Lhx8* expression was reduced in *Wnk1* knockout mice (red dot). (B–C) *Lhx8* expression by *in situ* hybridization. (B) Wild-type embryo at E10.5. (C) *Wnk1* knockout embryo at E10.5. The numbers of observed embryos was indicated. (D–I) *Awh* expression by *in situ* hybridization. (D–F) wild-type embryo. (G,H) embryos overexpressing *DWNK^D420A^* driven by *da*-*Gal4*. (I) *DWNK^EY18^* embryo. (D,G) stage 11, (E,H) stage 13, (F,I) stage 16. Lateral views. The numbers of embryos showing phenotypes and of total observed embryos were indicated. Anterior is left. Dorsal is up at stage 13 or 16. The detail genotypes in this figure were followings: (D–F) Canton-S (wild type): (G–H) *y w hsflp*; UAS-*DWNK^D420A^*/+; *da-Gal4*/+: (I) *y w hsflp*; *DWNK^EY18^* FRT2A.

The mammalian homologue of *Awh* is Lhx8 (also called L3 or Lhx7) [26]–[28]. To analyze how Lhx8 functions in the WNK signaling pathway, we first examined whether WNK1 binds to Lhx8 or regulates expression of the Lhx8 gene. We were unable to detect an interaction between ectopically expressed WNK1 and Lhx8 in cultured cells (data not shown). We performed microarray analyses using embryos of wild-type or *Wnk1* knockout mice at embryonic day 9. These microarray data revealed that *Lhx8* expression was reduced in *Wnk1* knockout mice (Fig. 4A). We also examined *Lhx8* expression in developing mice embryos by *in situ* hybridization. As previously reported [27], *Lhx8* was expressed in the craniofacial region of wild-type embryos at embryonic day 10.5 (Fig. 4B). In contrast, *Lhx8* expression was very weak in the similar region of *Wnk1* knockout mice embryos (Fig. 4C). Moreover, we examined whether *DWNK* controls *Awh* expression in *Drosophila* embryos. *Awh* was expressed in a striped pattern at stages 11 and 13 (Fig. 4D–E), and in the histoblast nest (which is the primordia of abdominal tissue) at stage 16 (arrows in Fig. 4F). We could not detect expression of *Awh* in the histoblast nests of embryos homozygous for *DWNK^EY18^* at stage 16 (Fig. 4I). However, expression of *Awh* in embryos homozygous for *DWNK^EY18^* at stages 11 and 13 was not changed (data not shown). We expected that the zygotic mutant embryos of *DWNK^EY18^* would be rescued by maternal transcripts of *DWNK*, since *DWNK* is maternally expressed according to the High Throughput Expression Data in Flybase (http://flybase.org). Instead, we found that expression of *Awh* at stages 11 and 13 was reduced in the embryos overexpressing *DWNK^D420A^* driven by *da-Gal4* (Fig. 4G–H). Together, these results suggest that the expression of the *Awh/Lhx8* gene is regulated by *WNK*.

### The WNK-SPAK/OSR1 pathway induces expression of the Lhx8 gene

**Figure 5:**
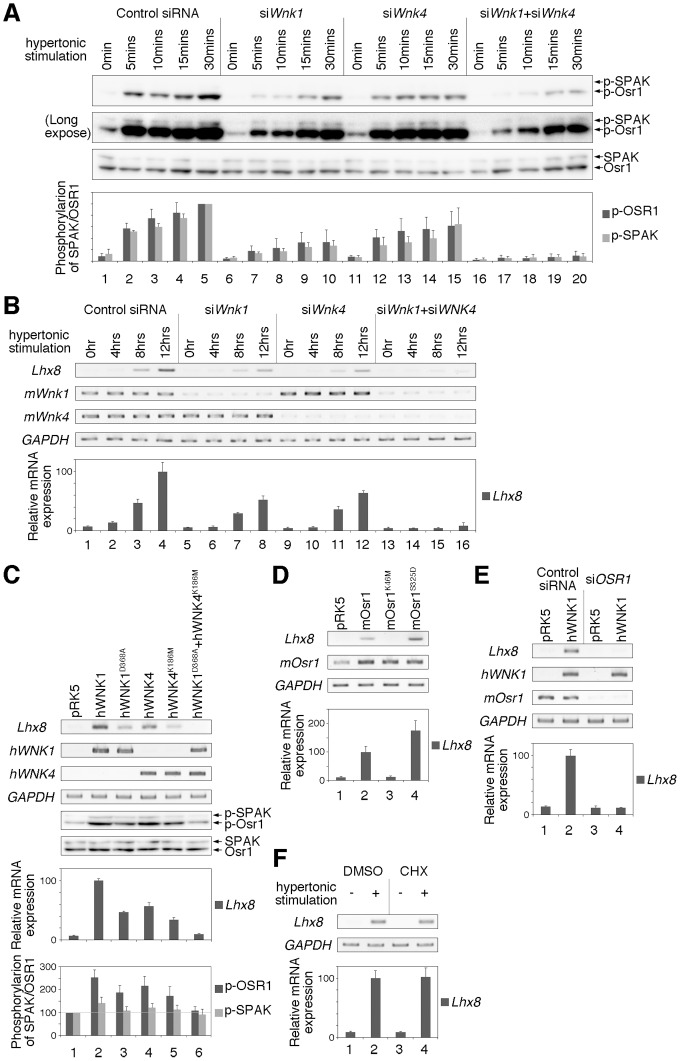
*Lhx8* is a downstream target of the WNK-OSR1 pathway. (A) Western blotting analysis of phospho-SPAK/OSR1, as an indicator of WNK activity in NIH3T3 cells. Upper panel showed the phosphorylation level of SPAK/OSR1. Middle panel showed the long exposed photo of the upper panel. Bottom panel shows the total protein of SPAK/OSR1. Cells were treated with siRNA; (lane 1–5) control siRNA, (lane 6–10) siRNA against *mWnk1* (si*Wnk1*), (lane 11–15) siRNA against *mWnk4* (si*Wnk4*), or (lane16–20) siRNA against both *mWnk1* and *mWnk4* (si*Wnk1*+si*Wnk4*). The value obtained from each samples was normalized to the level of SPAK or OSR1. The value of phospho-SPAK or OSR1 at 30 minutes from control siRNA (lane 5) was set to 100. (B) Gene expressions by RT-PCR or quantitative RT-PCR analysis were examined in NIH3T3 cells under hypertonic conditions. Cells were treated with siRNA; (lane 1–4) control siRNA, (lane 5–8) si*Wnk1*, (lane 9–12) si*Wnk4*, or (lane 13–16) si*Wnk1*+si*Wnk4*. The value obtained from each samples was normalized to the level of *GAPDH*. The value of *Lhx8* at 24 hours from control siRNA (lane 4) was set to 100. (C) Gene expressions by RT-PCR or quantitative RT-PCR analysis or Osr1 phosphorylation by Western blotting were examined in NIH3T3 cells overexpressing hWNK1 and/or hWNK4. The value obtained from each samples was normalized to the level of *GAPDH*, SPAK or OSR1. The value of *Lhx8*, phospho-SPAK or phospho-OSR1 from WNK1 expression (lane 2) was set to 100. (D) Gene expressions by RT-PCR or quantitative RT-PCR analysis were examined in NIH3T3 cells overexpressing mOsr1. The value obtained from each samples was normalized to the level of *GAPDH*. The value of *Lhx8* from mOsr1 expression (lane 2) was set to 100. (E) Gene expressions by RT-PCR or quantitative RT-PCR analysis were examined in NIH3T3 cells overexpressing hWNK1 and knockdown of *mOsr1* using siRNA. The value obtained from each samples was normalized to the level of *GAPDH*. The value of *Lhx8* from WNK1 expression under the treatment of control siRNA (lane 2) was set to 100. (F) Gene expressions by RT-PCR or quantitative RT-PCR analysis were examined in NIH3T3 cells under hypertonic condition with or without cycloheximide (CHX). The value obtained from each samples was normalized to the level of *GAPDH*. The value of *Lhx8* from control (lane 2) was set to 100.

We further attempted to examine *Lhx8* gene expression in the WNK-SPAK/OSR1 pathway. A previous study has reported that WNKs are activated by hypertonic stimulation [29]. We first performed Western blotting analysis using anti-phospho OSR1 antibody, which recognizes Ser325 of mOsr1 phosphorylated by WNK kinases^5^, whether WNKs are activated by hypertonic stimulation in NIH3T3 cells. We found that WNKs were immediately activated by hypertonic stimulation in NIH3T3 cells (Fig. 5A lanes 1–5). On the other hand, knockdown of either *Wnk1* or *Wnk4* using siRNA significantly reduced the phophorylation level of mOsr1 (Fig. 5A lanes 6–10 or 11–15). Moreover, the knockdown of both *Wnk1* and *Wnk4* synergistically caused the loss of the phosphorylation of mOsr1 (Fig. 5A lanes 16–20). Next, we performed RT-PCR analysis to ask whether the expression of *Lhx8* gene is activated under hypertonic conditions. We found that *Lhx8* expression was induced in NIH3T3 cells by hypertonic stimulation for 8 hours (Fig. 5B lanes 1–4). Knockdown of either *Wnk1* or *Wnk4* using siRNA resulted in significantly reduced induction of Lhx8 (Fig. 5B lanes 5–8 or 9–12). In addition, *Lhx8* activation was completely suppressed by knockdown of both *Wnk1* and *Wnk4* (Fig. 5B lanes 13–16). Conversely, *Lhx8* expression was induced by the expression of hWNK1 or hWNK4 (Fig. 5C lanes 2 and 4). Although expression of a kinase-dead form of either hWNK1 or hWNK4 (hWNK1^D368A^ or hWNK4^K186M^) also weakly activated *Lhx8* expression (Fig. 5C lanes 3 and 5), co-expression of both hWNK1^D368A^ and hWNK4^K186M^ did not activate *Lhx8* expression (Fig. 5C lane 6). Phosphorylation of Osr1 was also confirmed to correlate with the expression of Lhx8 (Fig. 5C bottom two rows). Moreover, expression of wild-type mOsr1 also induced *Lhx8* expression (Fig. 5D lane 2). The expression of mOsr1^K46M^, the kinase-dead form of mOsr1, did not induce *Lhx8* expression (Fig. 5D lane 3). Furthermore, expression of mOsr1^S325D^, the constitutively active form of mOsr1, strongly induced *Lhx8* expression (Fig. 5D lane 4). On the other hand, induction of *Lhx8* induced by expression of WNK1 was completely suppressed when *Osr1* was knocked down using siRNA (Fig. 5E lane 4). In addition, the prior treatment of cycloheximide (CHX), an inhibitor of protein biosynthesis, did not inhibit *Lhx8* expression by hypertonic stimulation (Fig. 5F), indicating that *Lhx8* is a direct target gene in WNK activation through hypertonic stimulation. These results suggest that the WNK-OSR1 pathway regulates *Lhx8* expression, and that Lhx8 is a downstream target gene in the WNK signaling pathway.

### The WNK signaling pathway is involved in neural specification

**Figure 6:**
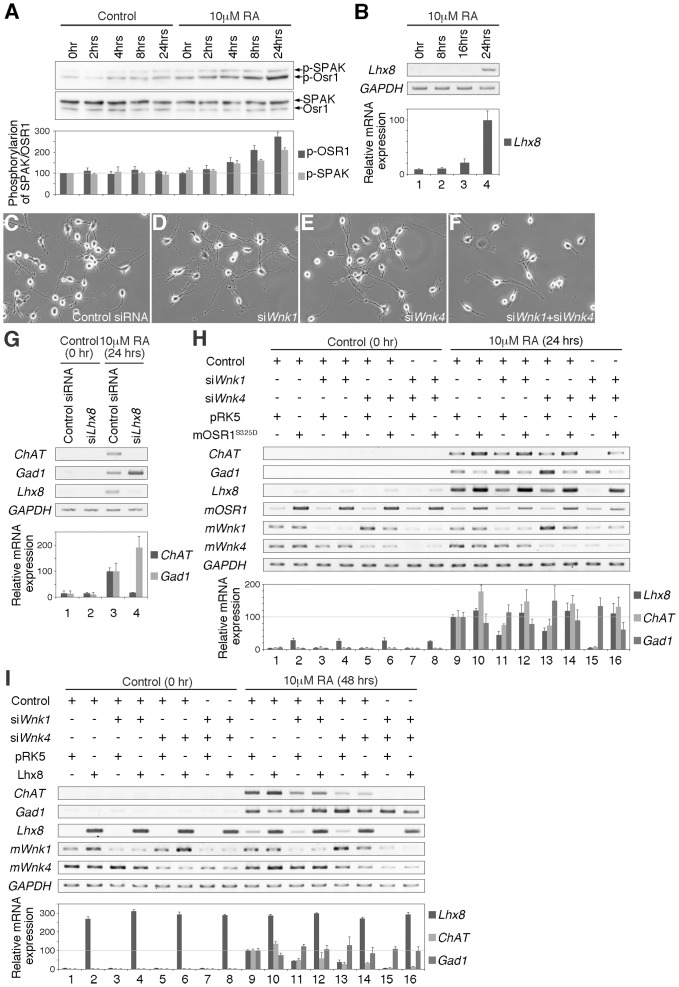
The WNK-OSR1-Lhx8 pathway is involved in the specification of neural fate. (A) Western blotting analysis of phospho-SPAK/OSR1, as an indicator of WNK signaling pathway activity in differentiating Neuro2A cells by retinoic acid (RA). The value obtained from each samples was normalized to the level of SPAK or OSR1. The value of phospho-SPAK or OSR1 at 0 minutes from undifferentiated cells (lane 1) was set to 100. (B) Expression of *Lhx8* induced by RA. The value obtained from each samples was normalized to the level of *GAPDH*. The value of *Lhx8* at 24 hours after induction (lane 4) was set to 100. (C–G) Differentiation of siRNA-treated Neuro2A cells induced by RA for 24 hours; (C) Control siRNA, (D) si*Wnk1*, (E) si*Wnk4* or (F) si*Wnk1*+si*Wnk4*. (G) Gene expressions by RT-PCR or quantitative RT-PCR analysis were examined in Neuro2A cells. Cells treated with siRNA against *Lhx8* (si*Lhx8*); (lanes 1–2) undifferentiated cells, (lanes 3–4) cells differentiated by RA for 24 hours. The value obtained from each samples was normalized to the level of *GAPDH*. The value of *ChAT* or *Gad1* from differentiated cells under the treatment of control siRNA (lane 3) was set to 100. (H–I) Neuro2A cells were transfected with various combinations of siRNAs and expression plasmids and gene expressions by RT-PCR or quantitative RT-PCR analysis were examined. Cells were treated with control siRNA (Control) in lanes 1,2,9,10, with si*Wnk1*in lanes 3,4,11,12, with si*Wnk4* in lanes 5,6,13,14, and with both *siWnk1 and siWnk4* in lanes 7,8,15,16. Cells were also transfected with control expression vector pRK5 in lanes 1,3,5,7,9,11,13,15 and with mOsr1^S325D^ (H) or Lhx8 (I) overexpression vector in lanes 2,4,6,8,10,12,14,16. Cells were left undifferentiated in lanes 1–8 and differentiated cells by RA for 24 hours (H) or 48 hours (I) in lanes 9–16. The value obtained from each samples was normalized to the level of *GAPDH*. The value of *Lhx8*, *ChAT* or *Gad1* from differentiated cells under the treatment of control siRNA (lane 9 in both H and I) was set to 100.

It is known that Lhx8 is involved in the determination of cholinergic neural fate in the forebrain [30], [31], and the specification of neural fate in Neuro2A cells that are able to differentiate into cholinergic or GABAergic neurons [32]. Moreover, WNK1 is known to play an important role in the proliferation, migration and differentiation of neural progenitor cells [12]. Thus, we speculated that the WNK signaling pathway might be involved in neural differentiation via expression of the *Lhx8* gene. Since retinoic acid (RA) is known to induce differentiation of Neuro2A cells, we investigated whether RA is able to activate WNK signaling. We performed Western blotting analysis using anti-phospho OSR1 antibody. We found that phosphorylation of mOsr1 increased at around 2 to 4 hours after RA stimulation (Fig. 6A), indicating that WNK kinases were activated by RA in Neuro2A cells. We also found that expression of the *Lhx8* gene was induced by RA in Neuro2A cells (Fig. 6B). After treatment with RA for 24 hours, Neuro2A cells were clearly differentiated and had generated several elongated neurites (Fig. 6C; see also Fig. S6). RA-induced neurite elongation was not suppressed by siRNA knockdown of either *Wnk1* or *Wnk4* alone in Neuro2A cells (Fig. 6D–E), but was inhibited by the combined knockdown of both *Wnk1* and *Wnk4* (compare Fig. 6F with Fig. 6C). Moreover, knockdown of *Lhx8* also inhibited neurite elongation (Fig. S6). These results suggest that the WNK signaling pathway is involved in the elongation of neurites.

To confirm the neural fate of the differentiated Neuro2A cells, we examined the expression of marker genes by RT-PCR analysis. *Choline acetyltransferase* (*ChAT*) or *Glutamic acid decarboxylase 1* (*Gad1*) was used as a marker for cholinergic or GABAergic neurons, respectively. After treatment of RA for 24 hours, Neuro2A cells differentiated into neurons and expressed the marker genes, *ChAT* and *Gad1* (Fig. 6G lane 3). As previously reported [32], we confirmed that knockdown of *Lhx8* expression caused a decrease in *ChAT* expression and increase in *Gad1* expression (Fig. 6G lane 4). Knockdown of *Wnk1* or *Wnk4* caused a decrease in *ChAT* and increase in *Gad1* expression, compared to cells treated with control siRNA (Fig. 6H lanes 9,11,13 and 6I lanes 9,11,13). In addition, knockdown of both *Wnk1* and *Wnk4* completely reduced *ChAT* expression (Fig. 6H lane 15 and 6I lane 15). These results suggest that the WNK kinases are involved in the specification of neural fate.

These results indicate that *Lhx8* expression correlates with the activities of the WNK kinases. Thus, we first examined whether expression of the constitutively active form of mOsr1 (mOsr1^S325D^) rescues the neural specification phenotype induced by knockdown of both *Wnk1* and *Wnk4*. While the expression of mOsr1^S325D^ did not affect and could rescue the elongation of neurite (Fig. S6), mOsr1^S325D^ overexpression significantly induced *Lhx8* expression even in undifferentiated Neuro2A cells (Fig. 6H lane 2,4,6,8) and also affected an enhancement in *ChAT* expression and a reduction in *Gad1* expression with RA treatment in Neuro2A cells (Fig. 6H lane 9–10). Under the condition of the knockdown of both *Wnk1* and *Wnk4*, mOsr1^S325D^ overexpression could rescue *Lhx8* expression, the decreasing of *ChAT* and increasing of *Gad1* (Fig. 6H lane 15–16). On the other hand, *Lhx8* overexpression did not affect the elongation of neurites (Fig. S6), but could not rescue the elongation of neurites caused by the knockdown of both *Wnk1* and *Wnk4* (Fig. S6). *Lhx8* overexpression also caused an enhancement in *ChAT* expression and a reduction in *Gad1* expression (Fig. 6I lane 9–10). However, *Lhx8* overexpression could not rescue the decrease in *ChAT* or increase in *Gad1* expression caused by knockdown of both *Wnk1* and *Wnk4* (Fig. 6I lane 15–16). These results suggest that WNK-OSR pathway is involved in the neural development, and that Lhx8 is necessary but not sufficient for the specification of the neural fate.

### The WNK signaling pathway is involved in neural development in *Drosophila*

**Figure 7:**
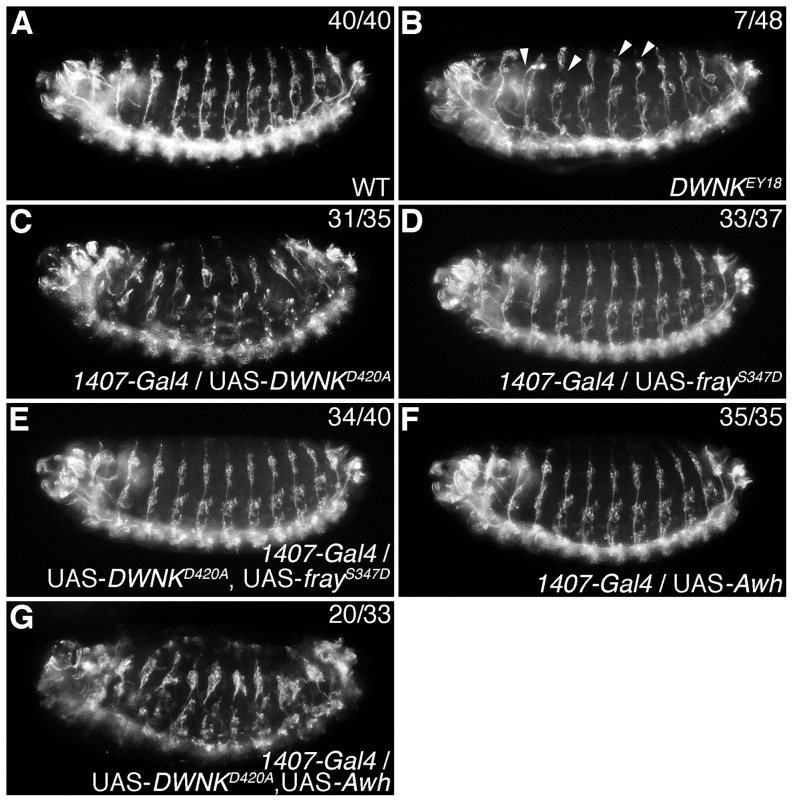
DWNK is important for neural development. (A–G) Lateral views of *Drosophila* embryos at stage 16 stained by 22C10 monoclonal antibodies. (A) Wild-type embryo. (B) *DWNK^EY18^* mutant embryo. Arrowheads indicate defects of axon guidances. (C) Embryos overexpressing *DWNK^D420A^* driven by *1407-Gal4*. (D) Embryos overexpressing *fray^S347D^* driven by *1407-Gal4*. (E) Embryos overexpressing *DWNK^D420A^* and *fray^S347D^* driven by *1407-Gal4*. (F) Embryos overexpressing *Awh* driven by *1407-Gal4*. (G) Embryos overexpressing *DWNK^D420A^* and *Awh* driven by *1407-Gal4*. The numbers of embryos showing phenotypes and of total observed embryos were indicated. Anterior is left. Dorsal is up. The detail genotypes in this figure were followings: (A) Canton-S (wild type): (B) *y w hsflp*; *DWNK^EY18^* FRT2A: (C) *y w hsflp*/*w*; UAS-*DWNK^D420A^*/*1407-Gal4*: (D) *y w hsflp*/*w*; *1407-Gal4*/+; UAS-*fray^S347D^*/+: (E) *y w hsflp*/*w*; UAS-*DWNK^D420A^*/*1407-Gal4*; UAS-*fray^S347D^*/+: (F) *y w hsflp*/*w*; *1407-Gal4*/+; UAS-*Awh*/+: (G) *y w hsflp*/*w*; UAS-*DWNK^D420A^*/*1407-Gal4*; UAS-*Awh*/+.

Since Fray was required for axonal ensheathment [24] and *Awh* was expressed in neuroblasts in stage 9 embryos in *Drosophila* [26], we wondered whether the WNK signaling pathway was also involved in neural development during *Drosophila* development. To analyze this possibility, we examined the formation of the peripheral nervous system in fly embryos by staining with 22C10 monoclonal antibodies, which can visualize neuronal morphology and axonal projections [33], [34] (Fig. 7A). We could find only a few *DWNK^EY18^* embryos exhibiting minor defects of axon guidance (arrows in Fig. 7B). These are expected that the zygotic mutant embryos of *DWNK^EY18^* would be rescued by maternal transcripts of *DWNK*, as explained above. Therefore to better confirm this result, we examined the effects of a dominant negative form of *DWNK*. We found that the expression of a dominant negative form of *DWNK* (*DWNK^D420A^*) driven by the *1407-Gal4* driver, which expressed in neuroblast and nervous system [35], also caused severe defects in the peripheral nervous system (Fig. 7C). We next tested whether *Fray* or *Awh* could rescue the phenotypes. While overexpression of the constitutively active form of *fray* (*fray^S347D^*) did not cause any phenotypes (Fig. 7D), the constitutively active form of *fray* could rescue the effects of the dominant negative form of *DWNK* (Fig. 7E). On the other hand, overexpression of *Awh* did not cause any defects of axon guidance (Fig. 7F), and could not rescue the phenotypes by overexpression of *DWNK^D420A^* (Fig. 7G). We also confirmed that the rescue by the co-expression of *fray^S347D^* was not due to the titration of *Gal4* expression (Fig. S5). These results suggest that DWNK-Fray pathway plays an important role in neural development in *Drosophila*, but that Awh is not sufficient for determinant in neural development.

## Discussion

The WNK-SPAK/OSR1 pathway is known to regulate various ion co-transporters and is widely conserved among many species [1], [2]. *Wnk1* knockout mice die before embryonic day 13 (Zambrowics et al. and in this report) [13], and display defects in cardiac development [14]. WNK1 is also required for cell division in cultured cells [11], and proliferation, migration and differentiation of neural progenitor cells [12]. Furthermore, PHAII patients display a number of other clinical features, such as an intellectual impairment, dental abnormalities and impaired growth in addition to hypertension [15]. Accordingly, the new role of the WNK signaling pathway described here may provide further insight into the development and pathogenesis of PHAII. In this study, we have identified Lhx8/Awh as a new downstream molecule in the WNK-SPAK/OSR1 pathway and discovered a novel function for the WNK-Lhx8 pathway in neural development.

There are four mammalian WNK family members, and WNK1 and WNK4 genes are linked to a hereditary form of human hypertension known as Pseudohypoaldosteronism type II (PHAII) [4]. In *Drosophila*, only one WNK gene, DWNK, has been identified. We found that both the wild-type and kinase-dead forms of WNK1 or WNK4 caused the up-regulation of *Lhx8* gene expression in NIH3T3 cells (Fig. 5D lanes 2–5). Similarly, our previous study showed that SPAK, a substrate of WNK1, was weakly phosphorylated by the kinase-dead form of WNK1 following a long incubation [5]. These results are inconsistent with the idea that the kinase-dead form of DWNK functions as a dominant-negative mutant in *Drosophila*. Studies of WNK1 and WNK4 suggest that these molecules phosphorylate each other and coordinated to regulate NaCl cotransport [36]. Therefore, these results raised the possibility that the kinase-dead forms of WNK1 and WNK4 coordinate with their respective endogenous WNK1 and WNK4 counterparts in mammalian cells. In fact, we found that co-expression of both kinase-dead forms of WNK1 and WNK4 did not cause either induction of *Lhx8* gene expression or phosphorylation of mOsr1 (Fig. 5D lane 6). These results suggest that the kinase activity of WNKs is required for induction of *Lhx8* gene expression and the activation of SPAK/OSR1, and that the kinase-dead form of WNK acts as an actual dominant-negative form in the signaling pathway. Furthermore, the expression of *Lhx8* by either hypertonic or RA stimulation was required for the expression of both WNK1 and WNK4 (Fig. 5B lanes 13–16, 6I lane 15 and 6J lane 15). Taken together, these results suggest that WNK1 and WNK4 function coordinately and redundantly in mammalian cells.

A previous report demonstrated that WNK1 might control the formation of microtubules in developing neurons [12]. On the other hand, other studies suggested that Lhx8 plays an important role in the development of basal forebrain cholinergic neurons [30], [31], [37], that Fray is required for axonal ensheathment [24], and that Awh is expressed in neuroblasts in stage 9 embryos in *Drosophila* [26]. In this study, we showed that the WNK-OSR1 pathway regulates *Lhx8* gene expression, that knockdown of both *Wnk1* and *Wnk4* in Neuro2A cells caused a shortening of neurites, as well as reduced *Lhx8* expression (Fig. 6G–J and Fig. S6), and that the expression of the constitutively active form of mOsr1, mOsr1^S325D^, could rescue the phenotype caused by the knockdown of both *Wnk1* and *Wnk4* (Fig. 6I and Fig. S6). In addition, mutation of *DWNK* or expression of a dominant-negative form of *DWNK* in fly embryos caused defects in axon guidance in the peripheral nervous system (Fig. 7B,C), and the constitutively active form of *fray*, *fray^S347D^*, expression could rescue the phenotypes by the expression of the dominant negative form of *DWNK* (Fig. 7E). Furthermore, ubiquitous expression of *Awh* by *da-Gal4* showed severe defects of axon guidance as similar to *DWNK^D420A^* expression by *da-Gal4* (Fig. S7), although neural specific expression of *Awh* did not showed any phenotype (Fig. 7F). Taken together, these findings clearly indicate that the WNK-OSR1/Fray-Lhx8/Awh pathway is involved in neural development. However, the phenotypes caused by knockdown of both *Wnk1* and *Wnk4*, such as the shortening of neurites and the reduction in *ChAT* expression, were not rescued by the expression of Lhx8 in Neuro2A cells (Fig. 6J and Fig. S6). In addition, the expression of *Awh* could not rescue the defects in the peripheral nervous system by the expression of the dominant-negative form of *DWNK* (Fig. 7G). Previous reports showed that Lhx8 might work with other factors, such as Lhx6 or Isl1 [27], [37], [38]. However, we also found that the expression of *Lhx6* and/or *Isl1* with Lhx8 could not rescue the defects by knockdown of both *Wnk1* and *Wnk4* in Neuro2A cells (Fig. S8). These results suggest that other molecule(s) are involved in neural differentiation induced by WNK signaling. Our studies may provide the first evidence identifying a target gene that acts downstream in the WNK-SPAK/OSR1 pathway, and demonstrate the significance of the WNK-OSR1-Lhx8 pathway in neural development. However, the details of how other unknown molecules controlled by WNK signaling specifically contribute to neural developmental remain to be determined and will require additional study.

Genetic mutations of *WNK1* or *WNK4* in PHAII patients result in abnormal expression of the *WNK1* gene or WNK4 kinase activity, respectively [4]. Abnormal activation of the WNK signaling pathway caused by these mutations result in the misregulation of NCCs in the kidney, which in turn causes hypertension [5]–[9], [39]. However, PHAII patients display other clinical features, such as an intellectual impairment, dental abnormalities and impaired growth [15]. Although these features are also thought to be caused by *WNK1* or *WNK4* mutations, the details of how these pathologies occur are unknown except for hypertension. In this study, we identified Lhx8 as a downstream target of the WNK signaling pathway (Fig. 5). We also found evidence that the WNK-Lhx8 pathway is involved in neural development (Fig. 6). Previous studies have shown that knockdown of *Lhx8* using antisense oligodeoxynucleotides caused the loss of tooth germ [40], and Lhx8 and Lhx6 are key regulators of mammalian dentition [41]. Furthermore, *Lhx8* knockout mice show a reduction in the number of cholinergic neurons in the ventral forebrain [30], [31], [37] and exhibit a severe deficit in spatial learning and memory [42]. These observations indicate that Lhx8 has essential functions in the formation of the tooth development, the specification of the cholinergic neurons and the processing of the spatial information in mice. Therefore, the similarities between the clinical features of PHAII and the phenotypes of *Lhx8* knockdown or knockout mice strongly suggest that the WNK-Lhx8 pathway is involved in the pathogenesis of PHAII, aside from hypertension. Further investigation will be needed to prove this hypothesis.

## Supporting Information

Figure S1 **WNK family proteins in human and fly, and the genetic map of** ***DWNK*** **locus.** (A) The homology among WNKs in humans and *Drosophila*. Red boxes indicate kinase domains. The percentages under the red boxes are the % homology to human WNK1. Green boxes indicate auto-inhibitory domains. Sky blue boxes are coiled-coil domains. Yellow boxes are acidic regions. (B) Genomic locus of the *Drosophila WNK* gene. pEY construct inserted into 1^st^ exon in EY10165 line, and the translational start site was deleted in *DWNK^EY18^* mutant. White boxes are untranslated regions. Black boxes are coding regions. Red line indicates the region deficient in the *DWNK^EY18^* mutant.
(TIF)

Figure S2 **Penetrance of wing phenotypes.** (A) Ratio of wing phenotypes in each genotype shown in Figure 2. We could observe delta phenotype at the tip of vein 4 (arrowheads in Figure 2) with or without extra veins around vein 5 (arrows in Figure 2).
(TIF)

Figure S3 **The phenotypes of** ***DWNK^D420A^*** **overexpression or** ***DWNK^EY18^*** **minute mosaic clones in wing or notum.** (A) Wing from *DWNK^D420A^* overexpressing flies driven by *sd*-*Gal4* showed the loss of wing margins. Arrowhead shows the loss of wing margin. Note that *DWNK^D420A^* overexpressing flies are raised at 20°C. Dorsal is up. Distal is right. (B–C) Wings with minute mosaic clones of *DWNK^EY18^* mutant showed the loss of wing margin or the extra vein. Arrowhead shows the loss of wing margin (B) and arrow shows the extra vein (C). Dorsal is up. Distal is right. Note that we didn't observe wing, which had both the loss of wing margin and the extra vein. The numbers of wings showing phenotypes and of total observed wings were indicated. (D) Dorsal view of adult notum with minute mosaic clones of *DWNK^EY18^* mutant showed the loss of both macro- and microchaetes. Thin black lines indicate the clone border. White arrows indicate the loss of microchaetes. White arrowheads indicate the loss of dorso-central bristles. Anterior is up. The number of notums showing phenotypes and of total observed notums were indicated, but we could not estimate a penetrance, since clones were randomly induced by heat shock. The detail genotypes in this figure were followings: (A) *w sd-Gal4*/+; UAS-*DWNK^D420A^*/+: (B–D) *y w hsflp*; *DWNK^EY18^* FRT2A/*hsGFP hsCD2*(*y^+^*) *M(3)i55 ri* FRT2A.
(TIF)

Figure S4 **The rescue of the abdominal phenotypes by** ***DWNK*** **mutant clones.** (A) Abdomen from adult with *DWNK^EY18^* minute clones and *DWNK* overexpression. *DWNK* was expressed only in *DWNK^EY18^* minute clones using the *Gal80* suppression technique. Thin black lines indicate the clone border (also *DWNK* expression area). Black arrows or black arrowheads show rescued abdominal cuticles or bristles, respectively. Dorsal views. Anterior is up. The detail genotype in this figure was followings: *y w* UAS-*DWNK*/*y w hsflp*; *arm*-*Gal4*/+; *DWNK^EY18^* FRT2A/*hsGFP hsCD2*(*y^+^*) *M(3)i55 Tub>Gal80* FRT2A.
(TIF)

Figure S5 **The titration of Gal4 lines.** (A–A′) Abdomen from pharate adult co-overexpressing *DWNK^D420A^* and GFP driven by *sd*-*Gal4*. Dorsal views. Anterior is up. (B–B″) Lateral views of *Drosophila* embryos co-overexpressing *DWNK^D420A^* and GFP driven by *1407-Gal4* at stage 16 stained by 22C10 monoclonal antibodies (pink) and anti-GFP antibodies (green). Anterior is left. Dorsal is up. The detail genotypes in this figure were followings: (A) *w sd-Gal4*/+; UAS-*DWNK^D420A^*/+; UAS-GFP/+: (B) *y w hsflp*/*w*; UAS-*DWNK^D420A^*/*1407-Gal4*; UAS-GFP/+.
(TIF)

Figure S6 **The phenotypes of the knockdown of** ***Lhx8*** **or the knockdown of** ***Wnk1*** **and/or** ***Wnk4*** **with or without concomitant** ***mOsr1^S325D^*** **or** ***Lhx8*** **overexpression in Neuro2A cells.** (A–B) The knockdown of *Lhx8* caused the shortening of neurites. Differentiation of siRNA-treated Neuro2A cells induced by retinoic acid (RA) for 24 hrs; (A) Control siRNA or (B) si*Lhx8*. (C–N) *mOsr1^S325D^* overexpression could, but *Lhx8* overexpression could not rescue the shortening phenotype of neurites by the knockdown of both *Wnk1* and *Wnk4*. Differentiation of siRNA-treated Neuro2A cells induced by RA for 24 hours (*mOsr1^S325D^*) or 48 hours (*Lhx8*) with or without concomitant *mOsr1^S325D^* or *Lhx8* overexpression; (C,G,K) Control siRNA, (D,H,L) siRNA against *mWnk1* (si*Wnk1*), (E,I,M) siRNA against *mWnk4* (si*Wnk4*), (F,J,N) both si*Wnk1* and si*Wnk4* (si*Wnk1*+si*Wnk4*), (C–F) with control vector (pRK5), (G–J) with *mOsr1^S325D^* expression vector or (K–N) with *Lhx8* expression vector.
(TIF)

Figure S7 **The neural defects by** ***da-Gal4*** **.** (A–B) Lateral views of *Drosophila* embryos at stage 16 stained by 22C10 monoclonal antibodies. Dorsal views. Anterior is up. (A) Embryos overexpressing *DWNK^D420A^* driven by *da-Gal4*. (B) Embryos overexpressing *Awh* driven by *da-Gal4*. The numbers of embryos showing phenotypes and of total observed embryos were indicated. Anterior is left. Dorsal is up. The detail genotypes in this figure were followings: (A) *y w hsflp*; UAS-*DWNK^D420A^*/+; *da-Gal4*/+: (B) *y w hsflp*; UAS-*Awh*/+; *da-Gal4*/+.
(TIF)

Figure S8 **Expression of Lhx6 and/or Isl1 with Lhx8 could not rescue the phenotypes by the knockdown of both** ***mWnk1*** **and** ***mWnk4*** **in Neuro2A cells.** (A–L) Lhx6 and/or Isl1 expression with Lhx8 expression could not rescue the shortening phenotype of neurites by the knockdown of both *Wnk1* and *Wnk4*. Differentiation of siRNA-treated Neuro2A cells induced by RA for 24 hours with or without concomitant Lhx6, Isl1 and/or Lhx8 expression; (A–F) Control siRNA, (G–L) both si*Wnk1* and si*Wnk4* (si*Wnks*), (A,G) with control vector (pRK5), (B,H) with Lhx6 expression vector, (C,I) with Isl1 expression vector, (D,J) with Lhx8 and Lhx6 expression vectors, (E,K) with Lhx8 and Isl1 expression vectors or (F,L) with Lhx8, Lhx6 and Isl1 expression vectors. (M) Gene expressions by RT-PCR or quantitative RT-PCR analysis were examined in Neuro2A. Cells treated with siRNA against both *mWnk1* and *mWnk4*; Cells were treated with control siRNA (Control) in lanes 1–6 and 13–18, with both *siWnk1 and siWnk4* (si*Wnks*) in lanes 7–12 and 19–24. Cells were also transfected with control expression vector pRK5 in lanes 1,7,13,19, with Lhx6 expression vector in lanes 2,8,14,20, with Isl1 expression vector in lanes 3,9,15,21, with Lhx8 and Lhx6 expression vectors in lanes 4,10,16,22, with Lhx8 and Isl1 expression vectors in lanes 5,11,17,23, or with Lhx8, Lhx6 and Isl1 expression vectors in lanes 6,12,18,24. (lanes 1–12) undifferentiated cells, (lanes 13–24) cells differentiated by RA for 24 hours. The value obtained from each samples was normalized to the level of *GAPDH*. The value of *ChAT* and *Gad1* from differentiated cells under the treatment of control siRNA (lane 13) was set to 100.
(TIF)

Figure S9 **The gel images of all PCR results.**
(TIF)
