# Supplementary figures and images for: WNK Signaling Is Involved in Neural Development via Lhx8/Awh Expression
Source: PLoS One. 2013 Jan 30;8(1):e55301. doi: 10.1371/journal.pone.0055301 (PMC3559379; doi:10.1371/journal.pone.0055301)

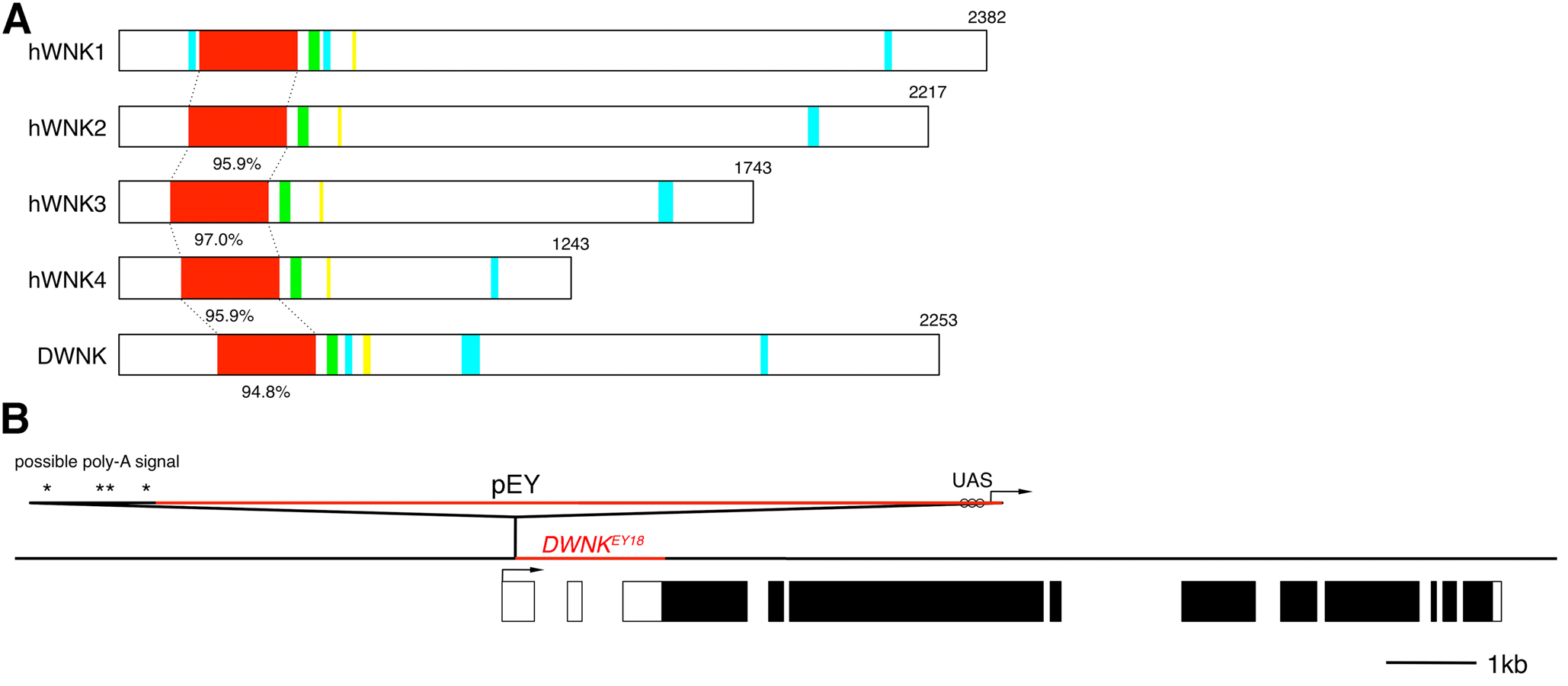

Supplement: Figure S1 — WNK family proteins in human and fly, and the genetic map of DWNK locus. (A) The homology among WNKs in humans and Drosophila. Red boxes indicate kinase domains. The percentages under the red boxes are the % homology to human WNK1. Green boxes indicate auto-inhibitory domains. Sky blue boxes are coiled-coil domains. Yellow boxes are acidic regions. (B) Genomic locus of the Drosophila WNK gene. pEY construct inserted into 1st exon in EY10165 line, and the translational start site was deleted in DWNKEY18 mutant. White boxes are untranslated regions. Black boxes are coding regions. Red line indicates the region deficient in the DWNKEY18 mutant. (TIF) [file pone.0055301.s001.tif]

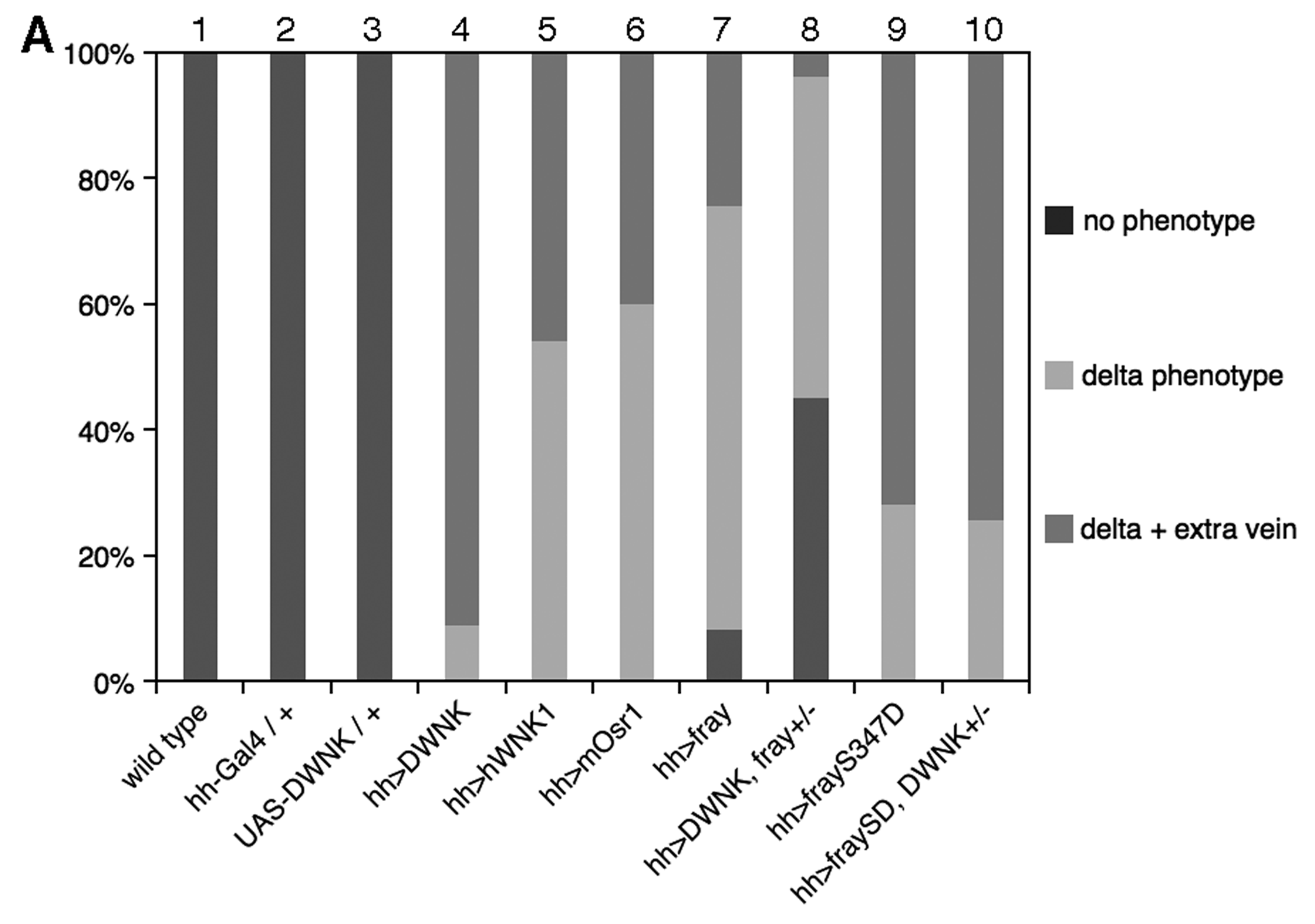

Supplement: Figure S2 — Penetrance of wing phenotypes. (A) Ratio of wing phenotypes in each genotype shown in Figure 2. We could observe delta phenotype at the tip of vein 4 (arrowheads in Figure 2) with or without extra veins around vein 5 (arrows in Figure 2). (TIF) [file pone.0055301.s002.tif]

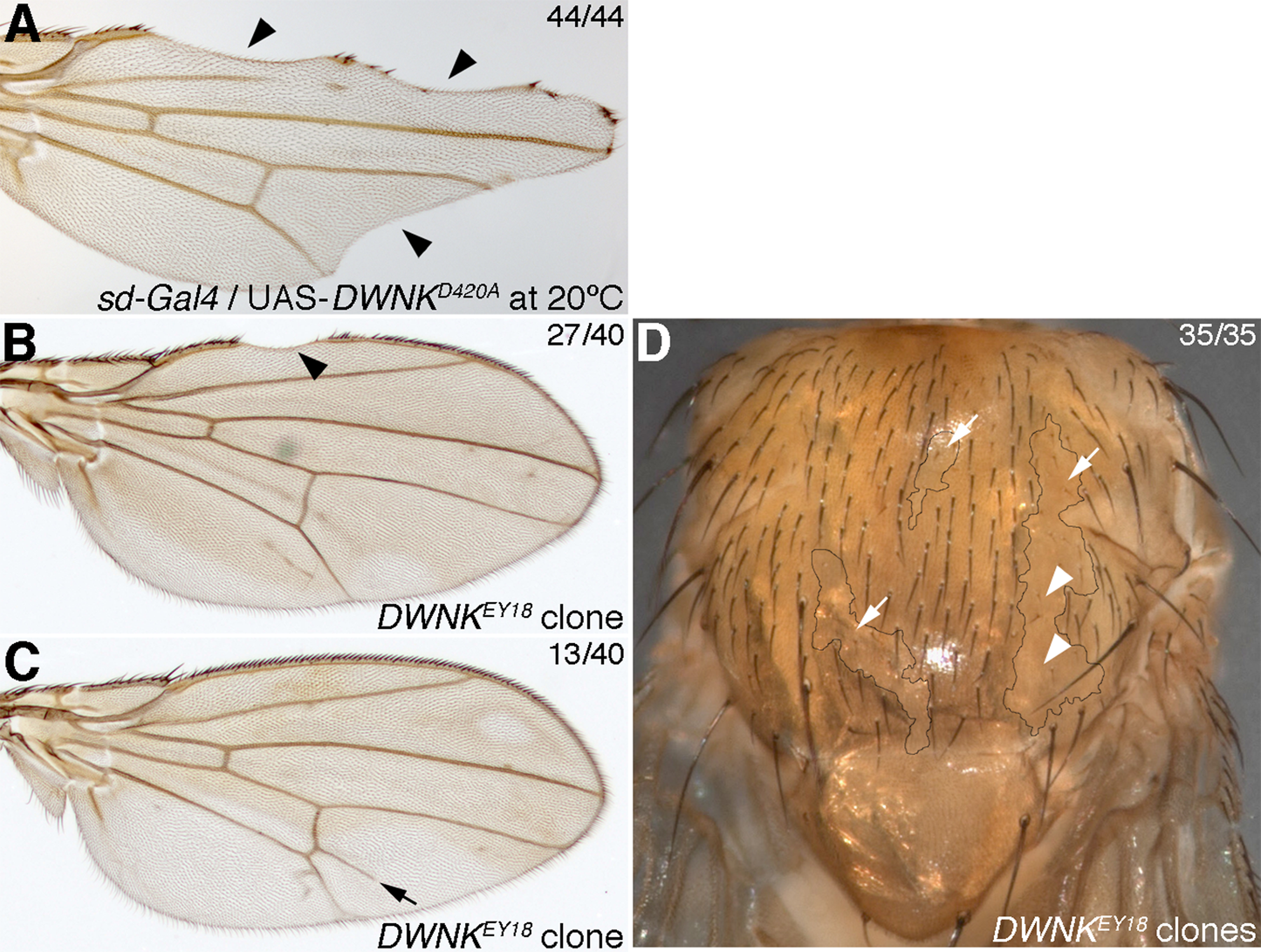

Supplement: Figure S3 — The phenotypes of DWNKD420A overexpression or DWNKEY18 minute mosaic clones in wing or notum. (A) Wing from DWNKD420A overexpressing flies driven by sd-Gal4 showed the loss of wing margins. Arrowhead shows the loss of wing margin. Note that DWNKD420A overexpressing flies are raised at 20°C. Dorsal is up. Distal is right. (B–C) Wings with minute mosaic clones of DWNKEY18 mutant showed the loss of wing margin or the extra vein. Arrowhead shows the loss of wing margin (B) and arrow shows the extra vein (C). Dorsal is up. Distal is right. Note that we didn't observe wing, which had both the loss of wing margin and the extra vein. The numbers of wings showing phenotypes and of total observed wings were indicated. (D) Dorsal view of adult notum with minute mosaic clones of DWNKEY18 mutant showed the loss of both macro- and microchaetes. Thin black lines indicate the clone border. White arrows indicate the loss of microchaetes. White arrowheads indicate the loss of dorso-central bristles. Anterior is up. The number of notums showing phenotypes and of total observed notums were indicated, but we could not estimate a penetrance, since clones were randomly induced by heat shock. The detail genotypes in this figure were followings: (A) w sd-Gal4/+; UAS-DWNKD420A/+: (B–D) y w hsflp; DWNKEY18 FRT2A/hsGFP hsCD2(y+) M(3)i55 ri FRT2A. (TIF) [file pone.0055301.s003.tif]

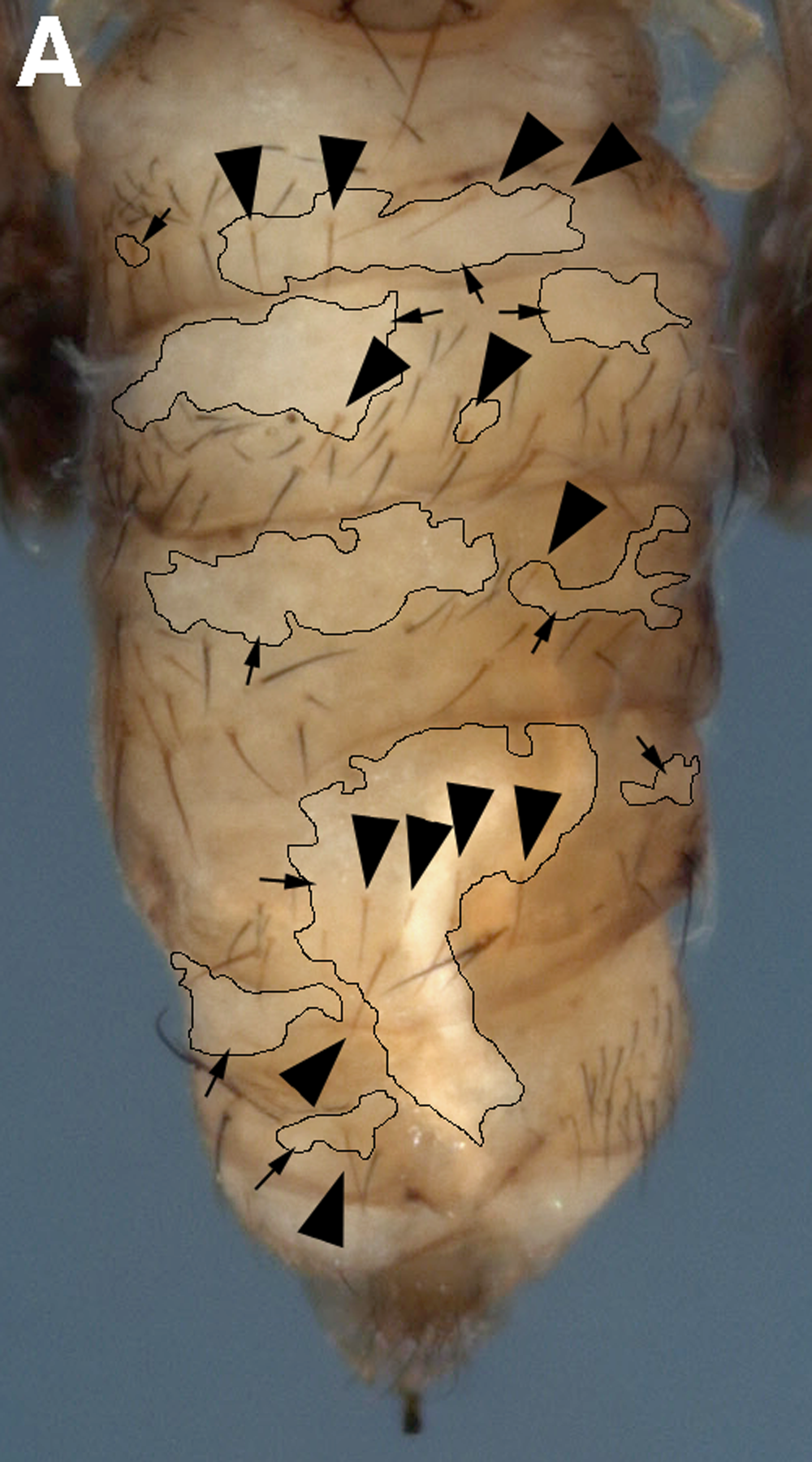

Supplement: Figure S4 — The rescue of the abdominal phenotypes by DWNK mutant clones. (A) Abdomen from adult with DWNKEY18 minute clones and DWNK overexpression. DWNK was expressed only in DWNKEY18 minute clones using the Gal80 suppression technique. Thin black lines indicate the clone border (also DWNK expression area). Black arrows or black arrowheads show rescued abdominal cuticles or bristles, respectively. Dorsal views. Anterior is up. The detail genotype in this figure was followings: y w UAS-DWNK/y w hsflp; arm-Gal4/+; DWNKEY18 FRT2A/hsGFP hsCD2(y+) M(3)i55 Tub>Gal80 FRT2A. (TIF) [file pone.0055301.s004.tif]

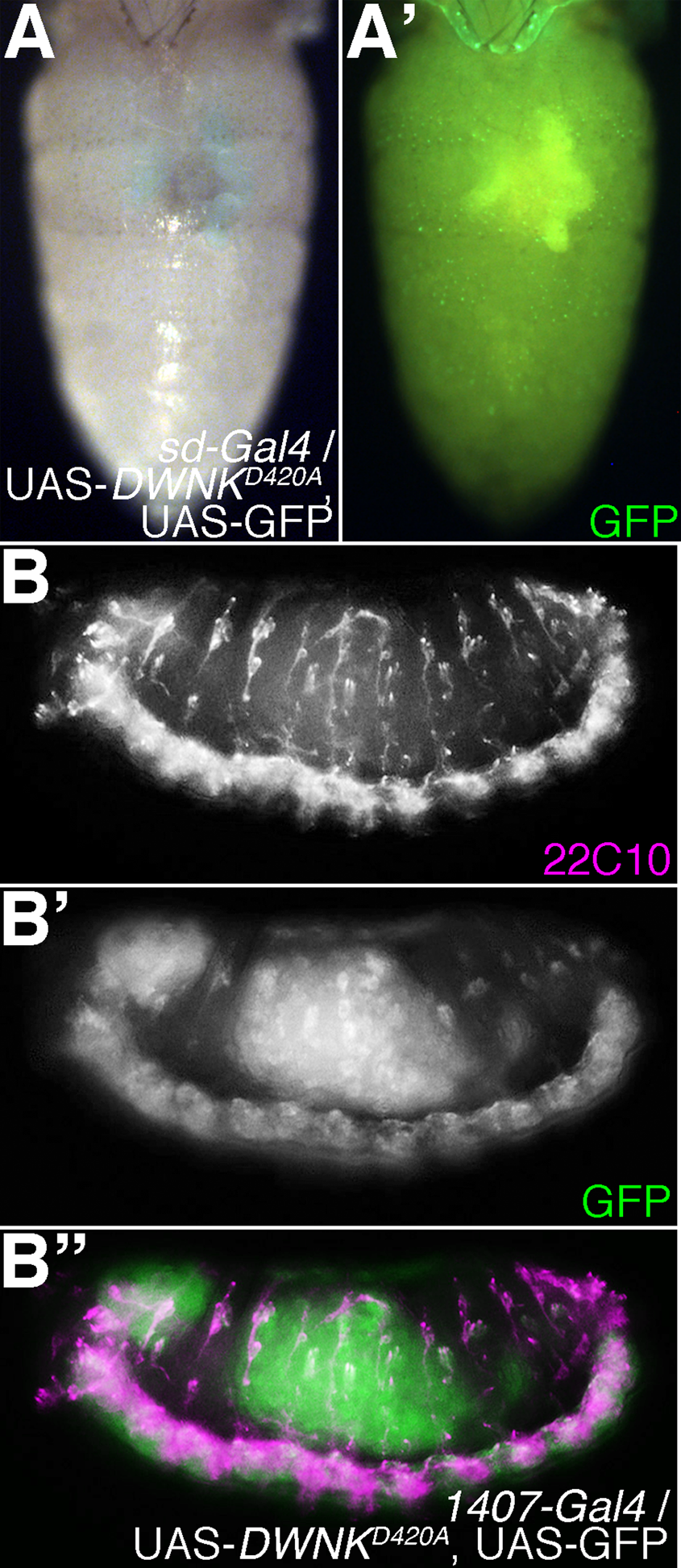

Supplement: Figure S5 — The titration of Gal4 lines. (A–A′) Abdomen from pharate adult co-overexpressing DWNKD420A and GFP driven by sd-Gal4. Dorsal views. Anterior is up. (B–B″) Lateral views of Drosophila embryos co-overexpressing DWNKD420A and GFP driven by 1407-Gal4 at stage 16 stained by 22C10 monoclonal antibodies (pink) and anti-GFP antibodies (green). Anterior is left. Dorsal is up. The detail genotypes in this figure were followings: (A) w sd-Gal4/+; UAS-DWNKD420A/+; UAS-GFP/+: (B) y w hsflp/w; UAS-DWNKD420A/1407-Gal4; UAS-GFP/+. (TIF) [file pone.0055301.s005.tif]

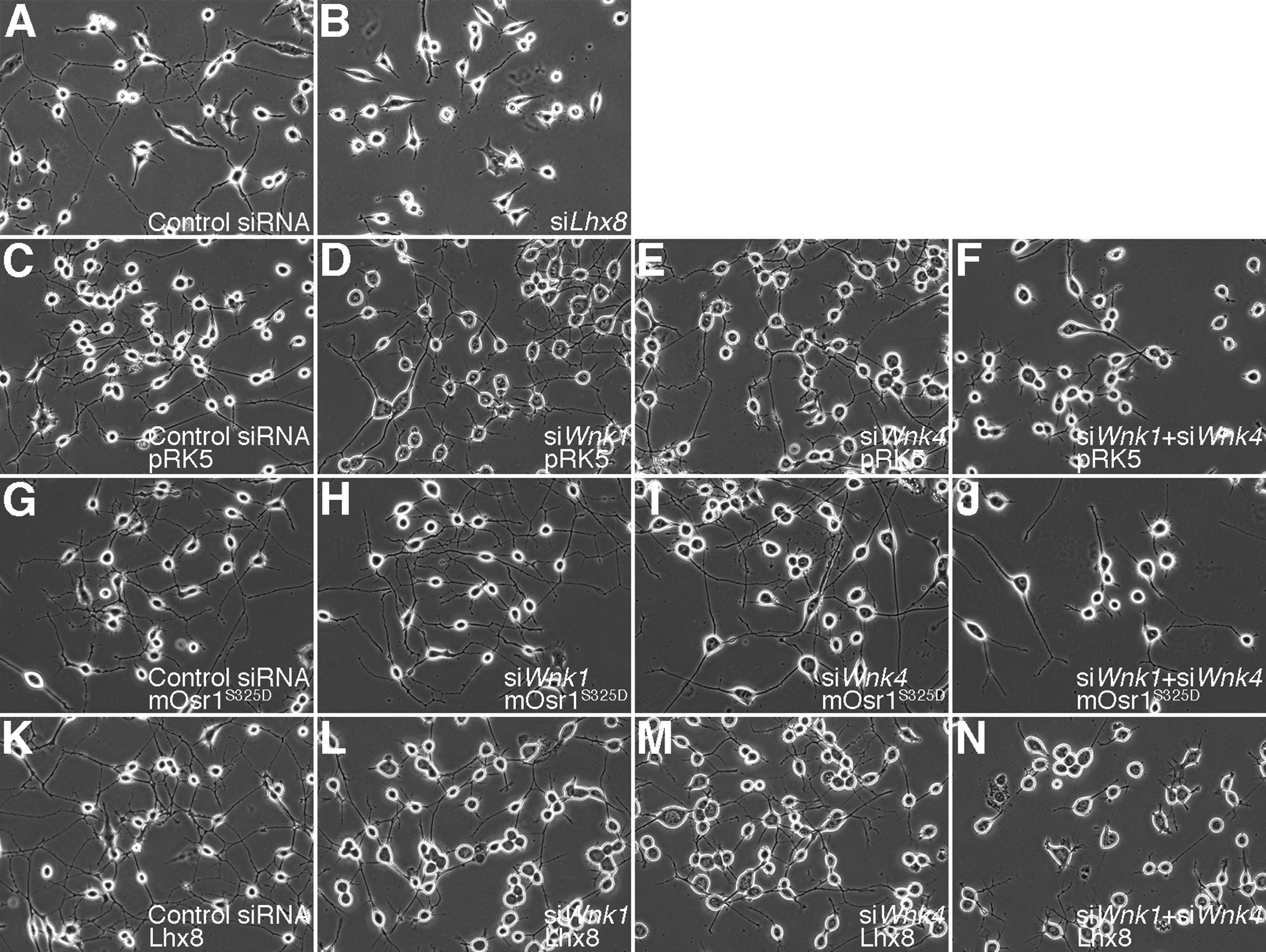

Supplement: Figure S6 — The phenotypes of the knockdown of Lhx8 or the knockdown of Wnk1 and/or Wnk4 with or without concomitant mOsr1S325D or Lhx8 overexpression in Neuro2A cells. (A–B) The knockdown of Lhx8 caused the shortening of neurites. Differentiation of siRNA-treated Neuro2A cells induced by retinoic acid (RA) for 24 hrs; (A) Control siRNA or (B) siLhx8. (C–N) mOsr1S325D overexpression could, but Lhx8 overexpression could not rescue the shortening phenotype of neurites by the knockdown of both Wnk1 and Wnk4. Differentiation of siRNA-treated Neuro2A cells induced by RA for 24 hours (mOsr1S325D) or 48 hours (Lhx8) with or without concomitant mOsr1S325D or Lhx8 overexpression; (C,G,K) Control siRNA, (D,H,L) siRNA against mWnk1 (siWnk1), (E,I,M) siRNA against mWnk4 (siWnk4), (F,J,N) both siWnk1 and siWnk4 (siWnk1+siWnk4), (C–F) with control vector (pRK5), (G–J) with mOsr1S325D expression vector or (K–N) with Lhx8 expression vector. (TIF) [file pone.0055301.s006.tif]

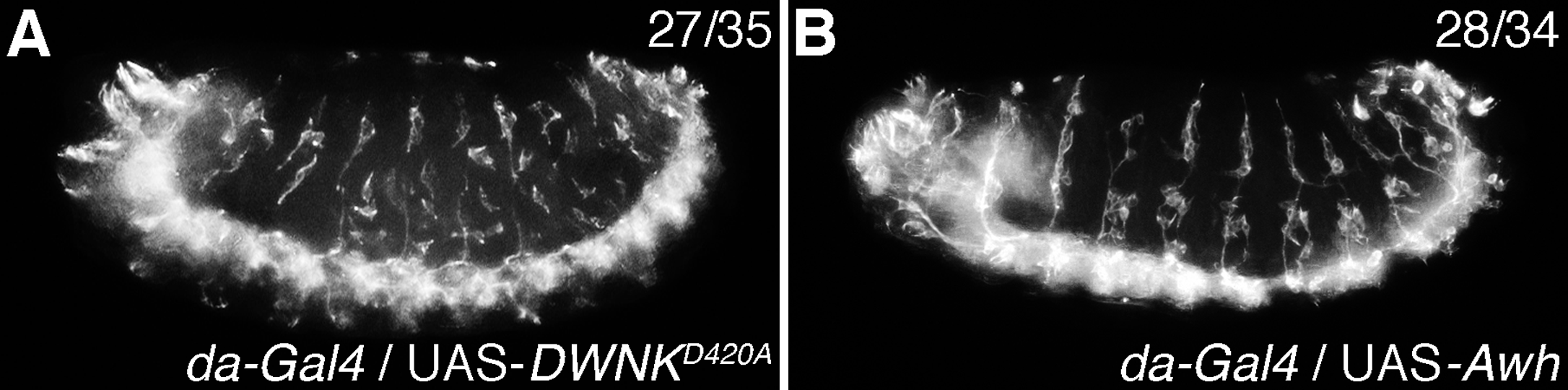

Supplement: Figure S7 — The neural defects by da-Gal4 . (A–B) Lateral views of Drosophila embryos at stage 16 stained by 22C10 monoclonal antibodies. Dorsal views. Anterior is up. (A) Embryos overexpressing DWNKD420A driven by da-Gal4. (B) Embryos overexpressing Awh driven by da-Gal4. The numbers of embryos showing phenotypes and of total observed embryos were indicated. Anterior is left. Dorsal is up. The detail genotypes in this figure were followings: (A) y w hsflp; UAS-DWNKD420A/+; da-Gal4/+: (B) y w hsflp; UAS-Awh/+; da-Gal4/+. (TIF) [file pone.0055301.s007.tif]

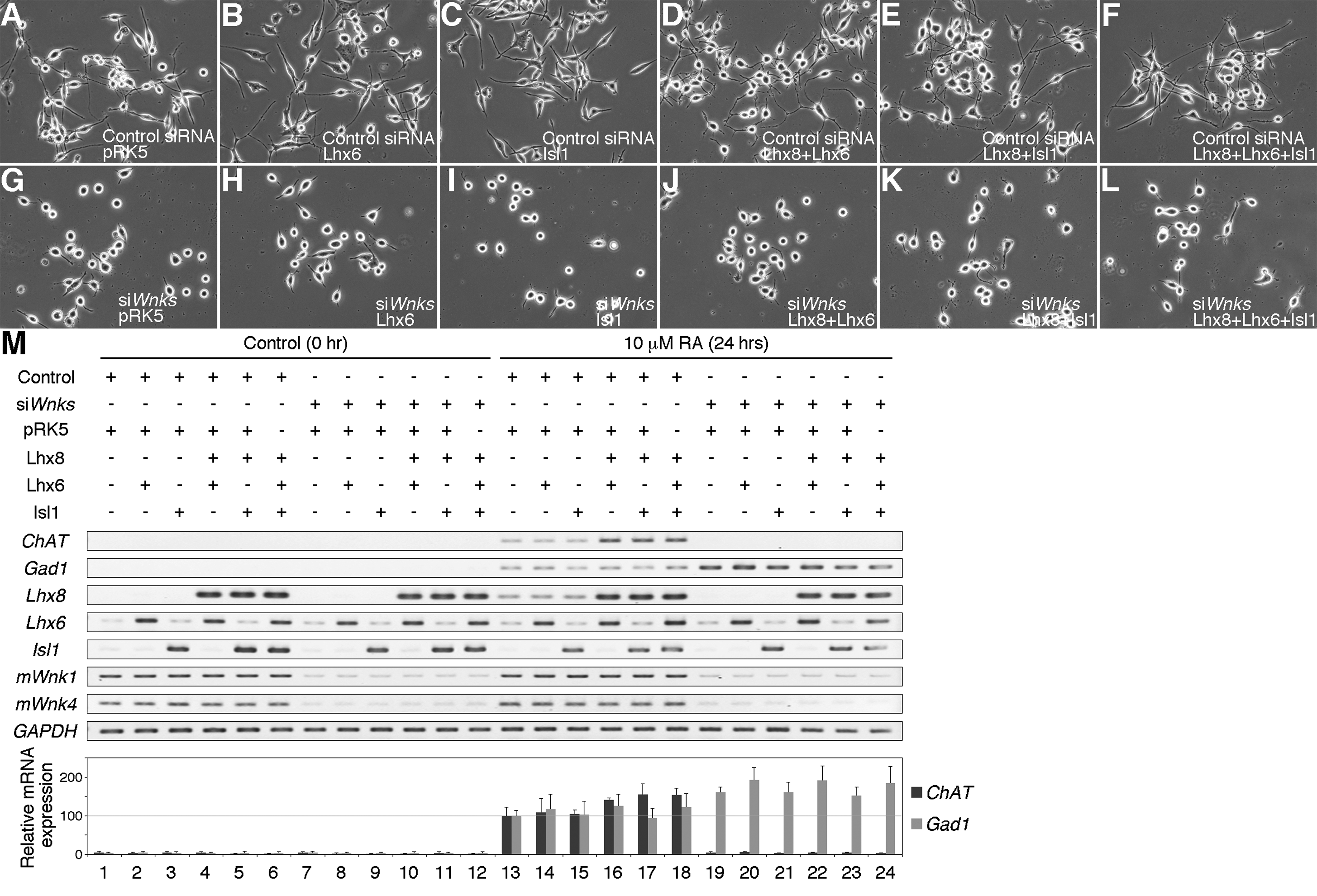

Supplement: Figure S8 — Expression of Lhx6 and/or Isl1 with Lhx8 could not rescue the phenotypes by the knockdown of both mWnk1 and mWnk4 in Neuro2A cells. (A–L) Lhx6 and/or Isl1 expression with Lhx8 expression could not rescue the shortening phenotype of neurites by the knockdown of both Wnk1 and Wnk4. Differentiation of siRNA-treated Neuro2A cells induced by RA for 24 hours with or without concomitant Lhx6, Isl1 and/or Lhx8 expression; (A–F) Control siRNA, (G–L) both siWnk1 and siWnk4 (siWnks), (A,G) with control vector (pRK5), (B,H) with Lhx6 expression vector, (C,I) with Isl1 expression vector, (D,J) with Lhx8 and Lhx6 expression vectors, (E,K) with Lhx8 and Isl1 expression vectors or (F,L) with Lhx8, Lhx6 and Isl1 expression vectors. (M) Gene expressions by RT-PCR or quantitative RT-PCR analysis were examined in Neuro2A. Cells treated with siRNA against both mWnk1 and mWnk4; Cells were treated with control siRNA (Control) in lanes 1–6 and 13–18, with both siWnk1 and siWnk4 (siWnks) in lanes 7–12 and 19–24. Cells were also transfected with control expression vector pRK5 in lanes 1,7,13,19, with Lhx6 expression vector in lanes 2,8,14,20, with Isl1 expression vector in lanes 3,9,15,21, with Lhx8 and Lhx6 expression vectors in lanes 4,10,16,22, with Lhx8 and Isl1 expression vectors in lanes 5,11,17,23, or with Lhx8, Lhx6 and Isl1 expression vectors in lanes 6,12,18,24. (lanes 1–12) undifferentiated cells, (lanes 13–24) cells differentiated by RA for 24 hours. The value obtained from each samples was normalized to the level of GAPDH. The value of ChAT and Gad1 from differentiated cells under the treatment of control siRNA (lane 13) was set to 100. (TIF) [file pone.0055301.s008.tif]

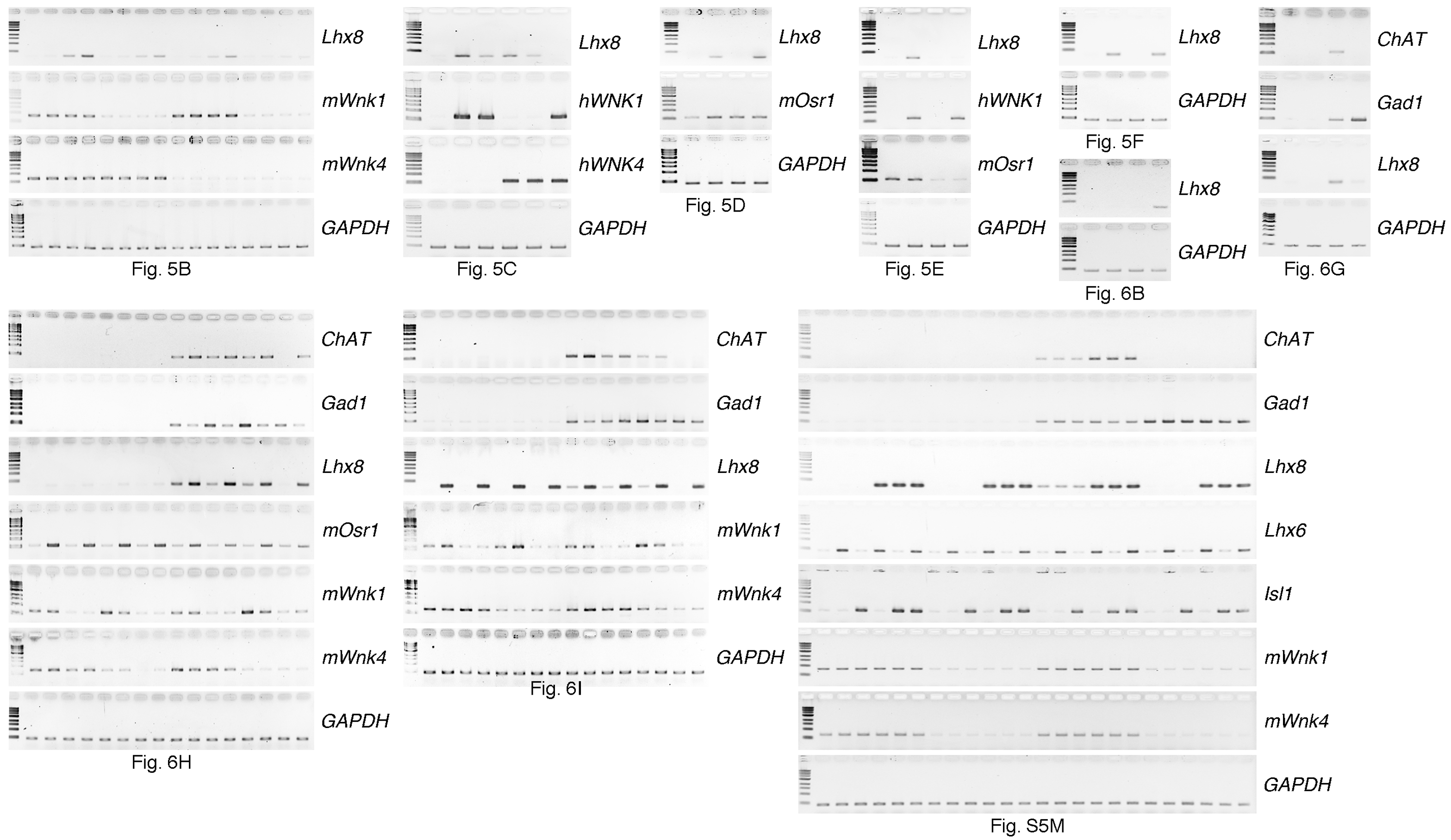

Supplement: Figure S9 — The gel images of all PCR results. (TIF) [file pone.0055301.s009.tif]
